# Neuropsychiatric Systemic Lupus Erythematosus Is Dependent on Sphingosine-1-Phosphate Signaling

**DOI:** 10.3389/fimmu.2018.02189

**Published:** 2018-09-26

**Authors:** Elise V. Mike, Hadijat M. Makinde, Evan Der, Ariel Stock, Maria Gulinello, Gaurav T. Gadhvi, Deborah R. Winter, Carla M. Cuda, Chaim Putterman

**Affiliations:** ^1^Department of Microbiology and Immunology, Albert Einstein College of Medicine, Bronx, NY, United States; ^2^Division of Rheumatology, Northwestern University School of Medicine, Chicago, IL, United States; ^3^Dominick P. Purpura Department of Neuroscience Animal Behavioral Core, Albert Einstein College of Medicine, Bronx, NY, United States; ^4^Division of Rheumatology, Albert Einstein College of Medicine, Bronx, NY, United States

**Keywords:** systemic lupus erythematosus, neuropsychiatric lupus, fingolimod, choroid plexus, RNA-seq

## Abstract

About 40% of patients with systemic lupus erythematosus experience diffuse neuropsychiatric manifestations, including impaired cognition and depression. Although the pathogenesis of diffuse neuropsychiatric SLE (NPSLE) is not fully understood, loss of brain barrier integrity, autoreactive antibodies, and pro-inflammatory cytokines are major contributors to disease development. Fingolimod, a sphingosine-1-phosphate (S1P) receptor modulator, prevents lymphocyte egress from lymphoid organs through functional antagonism of S1P receptors. In addition to reducing the circulation of autoreactive lymphocytes, fingolimod has direct neuroprotective effects such as preserving brain barrier integrity and decreasing pro-inflammatory cytokine secretion by astrocytes and microglia. Given these effects, we hypothesized that fingolimod would attenuate neurobehavioral deficits in MRL-lpr/lpr (MRL/lpr) mice, a validated neuropsychiatric lupus model. Fingolimod treatment was initiated after the onset of disease, and mice were assessed for alterations in cognitive function and emotionality. We found that fingolimod significantly attenuated spatial memory deficits and depression-like behavior in MRL/lpr mice. Immunofluorescent staining demonstrated a dramatic lessening of brain T cell and macrophage infiltration, and a significant reduction in cortical leakage of serum albumin, in fingolimod treated mice. Astrocytes and endothelial cells from treated mice exhibited reduced expression of inflammatory genes, while microglia showed differential regulation of key immune pathways. Notably, cytokine levels within the cortex and hippocampus were not appreciably decreased with fingolimod despite the improved neurobehavioral profile. Furthermore, despite a reduction in splenomegaly, lymphadenopathy, and circulating autoantibody titers, IgG deposition within the brain was unaffected by treatment. These findings suggest that fingolimod mediates attenuation of NPSLE through a mechanism that is not dependent on reduction of autoantibodies or cytokines, and highlight modulation of the S1P signaling pathway as a novel therapeutic target in lupus involving the central nervous system.

## Introduction

Systemic lupus erythematosus (SLE) is a chronic autoimmune disease characterized by the presence of autoreactive B and T lymphocytes, the overproduction of autoreactive antibodies, and tissue deposition of immune complexes. Worldwide prevalence ranges from 20 to 70 per 100,000 (1), and the disease preferentially affects women of Afro-Caribbean, Asian, and Hispanic descent (2). SLE results in multiple end-organ pathologies, including skin disease, arthritis, and renal disease that is a major cause of morbidity and mortality (3). Approximately 40% of SLE patients present with neurological or psychiatric symptoms that precede or may occur independently of active systemic disease and significantly decrease quality of life (4). These neuropsychiatric lupus (NPSLE) manifestations include a wide array of clinical presentations that can be broadly categorized as focal or diffuse (5). While focal symptoms such as seizure and stroke are known to be caused by underlying vasculopathy or thromboembolism, the pathophysiology of diffuse neuropsychiatric manifestations, including cognitive dysfunction, memory loss, and affective disorders, is not fully understood.

One important consequence from the lack of a clear understanding of the pathogenesis of diffuse NPSLE is the use of treatments that are generally non-curative or purely aimed at symptom control (4). Patients are frequently prescribed broad immunosuppression, and these medications often result in a host of deleterious side effects. Optimally, therapies should instead be focused on treating the underlying mechanisms, but further research would be required to define more targeted treatment regimens (4). More recent studies have therefore been directed toward therapeutic modulation of the cellular and cytokine microenvironment to affect downstream pathways and quell neuroinflammation.

Fingolimod, a sphingosine-1-phosphate (S1P) receptor modulator, is an immunosuppressant that results in lymphocyte sequestration. S1P is a bioactive signaling sphingolipid that is expressed at micromolar concentration in blood and nanomolar concentration in interstitial fluid of tissues (6), creating a gradient that is essential for naïve and central memory T cells to overcome the CCR7 retention signal and egress from the lymph node (7). After phosphorylation by sphingosine kinases, fingolimod functionally antagonizes S1P1 receptors expressed on lymphocytes, resulting in receptor internalization and degradation. This process prevents a functionally normal response to the endogenous S1P gradient, thereby blocking lymphocyte egress from lymphoid organs to the bloodstream and reducing the circulation of autoreactive lymphocytes (8).

Infiltration of the central nervous system (CNS) by autoreactive lymphocytes is believed to play an integral role in the neuroinflammation observed in murine models of NSPLE (9), much like T cell destruction of myelin is a critical aspect of multiple sclerosis (MS) pathogenesis (10). Fingolimod exerts robust therapeutic effects in experimental autoimmune encephalitis (EAE), an animal model of MS, and its efficacy at different stages of disease has been extensively reported (11). Therapeutic administration of fingolimod decreases clinical symptoms such as paralysis and motor coordination deficits and reduces pathological manifestations including demyelination and inflammatory infiltrates, even when administered at late stages of disease (12–15). Fingolimod has also proven to be effective prophylactically, preventing the development of EAE pathology (15).

Fingolimod's success in animal models of MS led to several clinical trials that eventually demonstrated its safety and efficacy in MS patients. It is currently the only oral FDA-approved treatment for relapsing-remitting MS, and a 2-year study revealed that patients had 52% fewer relapses while taking fingolimod (16). Further, fingolimod significantly slows disease progression and reduces ring-enhancing lesions (16).

In addition to the well-characterized effect on lymphocytes, lipophilic fingolimod readily crosses the blood brain barrier (BBB). After onset of EAE in rodents, uptake of fingolimod into the CNS after oral administration increased in a time- and dose-dependent manner (17). Moreover, both fingolimod and its active metabolite, fingolimod-phosphate, demonstrate several-fold higher concentration in the brain than in blood. This demonstrates the ability of endogenous sphingosine kinases within the CNS to convert fingolimod to its active charged form (17). In humans, a radiolabeled fingolimod analog, BZM055, exhibited brain uptake that increased over time after intravenous administration (18, 19). Given its efficacy in MS treatment, fingolimod is believed to act directly upon several cell types within the CNS that express S1P receptors (20). Cell types that have been implicated in NPSLE pathogenesis, including microglia (21, 22), endothelial cells (23), astrocytes (24) and neurons (25), exhibit a neuroprotective phenotype upon fingolimod treatment.

While several spontaneous SLE mouse models develop some cognitive or affective deficits, they are not all equally suited for the investigation of NPSLE pathogenesis. BXSB mice exhibit learning deficits (26), but the male predominance of this phenotype does not parallel human disease. NZB/W F1 mice exhibit neuroinflammation (27) and a cognitive and affective phenotype, but not as robust as in MRL/lpr mice (28). Additionally, both the BXSB and NZB/W F1 possess structural abnormalities that potentially confound the neurobehavioral profile seen in these mice (29, 30). MRL/lpr mice exhibit spatial memory deficits and depression-like behavior, starting at an early age (31). Further, they manifest autoreactive antibodies, neuroinflammation, and brain barrier disruption, all of which are believed to play a role in diffuse NPSLE pathogenesis (30). Therefore, the MRL/lpr mouse remains the most robust model for the examination of diffuse NPSLE, and provides a system better suited for therapeutic investigation.

Fingolimod's ability to reduce glial cytokine production, improve BBB integrity, and confer neuroprotection make it an attractive potential therapy for neuroinflammatory diseases. However, the effects of fingolimod treatment in a validated model of NPSLE have yet to be determined. Given its efficacy in reducing neuroinflammation and BBB disruption in an autoimmune neuroinflammatory disease, we hypothesized that fingolimod-mediated S1P receptor modulation would ameliorate NPSLE. We therefore investigated its effects on behavioral manifestations in the MRL/lpr mouse, an extensively validated and the most commonly used NPSLE model with many important parallels to human disease (31).

## Materials and methods

### Mice

Female MRL-Fas lpr/lpr (MRL/lpr) mice (stock #485) were purchased from The Jackson Laboratory (Bar Harbor, Maine) at 3 weeks of age and housed 4 mice per cage at a temperature of 21–23°C on a 12:12 h light:dark cycle. All animal study protocols were approved by the Institutional Animal Care and Use Committee at the Albert Einstein College of Medicine.

### Experimental design

Two cohorts of 10-week-old MRL/lpr mice (*n* = 16 in each cohort) were each randomized into 2 groups and received intraperitoneal injection of either 3 mg/kg fingolimod dissolved in saline (*n* = 8) or vehicle control (*n* = 8) three times per week, starting at 10 weeks of age. Serum was collected via retro-orbital bleed every 2 weeks and urine was collected weekly to assess the progression of autoantibody development and proteinuria, respectively. Two cohorts of mice were evaluated by behavioral testing after 4 weeks of treatment. Importantly, we could not perform a baseline exam as well since initial testing can significantly interfere with subsequent analysis of murine behavior patterns. After sacrifice, spleen and lymph node weight were determined and brains were either fixed, paraffin-embedded, and sectioned for histological analysis, or dissected and snap-frozen in liquid nitrogen and stored at−80°C for analysis of gene and protein expression. Brains from a separate cohort of mice (*n* = 10/group) were evaluated by flow cytometry to assess cell distribution and to sort individual populations for RNA sequencing and downstream analysis.

### Assessment of systemic disease

Serum IgG autoantibodies against double-stranded (ds)DNA were measured by ELISA. Plates were coated with 0.1 mg/mL salmon sperm DNA (ThermoFisher Scientific, Waltham, MA) in PBS in a warm room overnight and then rinsed with PBS the next day. Plates were blocked with 1% BSA in PBS for 1 h at 37°C. Samples were added at a 1:100 dilution and 6 subsequent two-fold dilutions to 1:3200, and incubated for 2 h at 37°C. After washing, samples were incubated with an alkaline phosphatase conjugated goat anti-mouse secondary antibody (Southern Biotech) and incubated for 1 h at 37°C. The plate was then washed, developed with phosphatase substrate (Sigma Aldrich) in 1M diethanolamine, pH 9.8 (Sigma Aldrich), and read at 405 nm using an Infinite F50 microplate reader (Tecan Group, Switzerland). Absorbance is reported as (optical density of sample/optical density of positive control 20-week old MRL/lpr serum on the same plate) x 100. Proteinuria was assessed semiquantitatively via Uristix where +1 is 30 mg/dL, +2 is 100 mg/dL, +3 is 300 mg/dL, and +4 is 2,000 mg/dL (Siemens Healthcare, Tarrytown, NY).

### Behavioral assessment

A series of tests extensively validated and employed in MRL/lpr mice (31–34) were performed to assess cognition and depression. The behavioral spectrometer was used to assess general locomotor activity, grooming, rearing, and exploration of the center zone (35). Additional tests included the Porsolt swim test for immobility (36), the object placement test for spatial memory, and the object recognition test for recognition memory (37). Mice were equilibrated in the test room for 30 min prior to the tests under low incandescent light with soft background noise. All four assessments were employed after 4 weeks of treatment. The behavioral spectrometer, object placement test, and object recognition tests were repeated after 8 weeks of treatment with new objects when applicable, while the forced swim test could not be repeated because of habituation to test conditions. All tests were digitally recorded by Viewer tracking software (Biobserve, Bonn, Germany).

#### Behavioral spectrometer

The behavioral spectrometer (Behavioral Instruments: Hillsborough, NJ) is a 40 × 40 cm enclosed arena with a vibration sensitive floor, infrared beams, and video tracking to detect and measure behavior. Mice were placed in the behavioral spectrometer for 9 min, and behaviors were measured using Viewer Software. Stereotyped behaviors, including rearing, sniffing, and grooming, as well as track length, time spent in the center of the arena, and number of center entries were assessed (35). The central zone was defined as a 15 × 15 cm area in the center of the box.

#### Object placement test

The object placement test is based on the tendency of mice to preferentially explore objects in novel positions as previously described (37). Briefly, mice were placed in a 40 × 40 cm arena and given a training phase of 5 min to freely explore two identical objects. Mice were then returned to their home cage for a 20-min retention interval and subsequently placed back in the arena in which one of the objects had been relocated to a new position. The amount of time that mice spent exploring the two objects during the 3-min testing interval was measured manually with a stopwatch by an observer blind to the condition of the mice. Exploration was defined as time spent in contact with the object. The preference score, or percent preference, was defined as (the amount of time spent exploring the object in the new position/total exploration time) × 100. A preference score of >55% for the object in the new position was considered “passing” (38). Training and testing phases were recorded by Viewer software. The total exploration time during the training phase was also measured in order to ensure that mice in all conditions had equivalent levels of exploration. Mice with <3 seconds of total exploration during the training phase were excluded.

#### Object recognition test

The object recognition test is also based on the robust tendency of mice to preferentially explore novel objects as previously described (37). In this task, mice are again placed in the arena with two identical objects for 3 min for a training phase. The retention interval used was 45 min. During the testing phase, a new object of similar visual richness was introduced in the same location as one of the training objects to evaluate recognition memory, and the number of seconds mice spent exploring both objects was measured. Objects were previously validated to ensure equal exploratory valence and were counterbalanced during training and testing. A preference score, or percent preference, of >55% for the novel object was considered passing. Exploration during the training phase was also measured, and both training and testing phases were recorded by Viewer software. The total exploration time during the training phase was also measured in order to ensure that mice in all conditions had equivalent levels of exploration. Mice with <3 seconds of total exploration during the training phase were excluded.

#### Porsolt forced swim test

Mice were placed individually in a beaker of water maintained at 25°C for 10 min and recorded using Viewer software. Mice tend to swim and climb when immersed in water, and immobility during this task is indicative of behavioral despair, i.e., depression-like behavior. The time spent immobile was scored manually for 3 consecutive 3-min time bins. Immobility was reported as % immobility: (time spent immobile/total time) x 100.

### Immunofluorescence

At 20 weeks of age, mice were transcardially perfused first with ice cold PBS and then with 2.5% paraformaldehyde. Brain tissue was isolated and further fixed in 2.5% paraformaldehyde for 48 h. Brain tissue was embedded in paraffin for coronal sectioning. For immunofluorescent staining, sections were deparaffinized in xylene and rehydrated in ethanol. Antigen retrieval was performed in citrate or Tris-EDTA buffer and sections were blocked in 20% normal horse serum. Staining for Iba-1 (rabbit anti-Iba-1, Wako, Osaka, Japan), IgG (donkey anti-mouse IgG, Jackson Immunoresearch Laboratories, West Grove, PA), CD3 (rabbit anti-mouse CD3, ThermoFisher Scientific), CD4 (rabbit anti-mouse CD4, Sino Biological, Wayne, PA), CD8 (rat anti-mouse CD8, eBioscence, San Diego, CA), B220 (rat anti-mouse B220, BD Biosciences, San Jose, CA), and albumin (goat anti-mouse albumin, Bethyl Laboratories, Montgomery, TX), were performed on 5 μm coronal sections. All fluorophore-conjugated secondary antibodies were from Jackson ImmunoResearch, West Grove, PA. Following immunostaining, all sections were counterstained with DAPI, washed, air dried, and mounted. Primary and secondary antibodies were withheld from sections to assess background and non-specific staining. Images were taken on a fluorescent microscope (Evos FL Auto 2, ThermoFisher Scientific) and quantified using ImageJ (National Institutes of Health, Bethesda, Maryland, USA). CD4+ and CD8+ T cells, B220+ B cells, and Iba-1+ macrophages were counted using the ImageJ cell counter plugin. Albumin leakage and IgG deposition were calculated as the mean fluorescent intensity in regions of interest to determine an intensity score.

### Histopathological scoring

Coronal brain sections stained with hematoxylin and eosin (H&E) were blindly scored for lymphocytic infiltration. The following scoring system was used: 0 = no infiltration, 1 = few individual cells, no clustering or stromal expansion, 2 = clusters of cells with or without stromal expansion, 3 = clusters of 8-12 cells with stromal expansion, 4 = multiple large clusters of over 12 cells each with stromal expansion.

### Tissue processing, flow cytometry, and fluorescence activated cell sorting (FACS)

Mice were transcardially perfused first with ice cold PBS. Brains were then excised and placed in ice cold HBSS until processing. The brains were weighed, infused with digestion buffer [2.5 mg/mL Liberase TL (Roche, Basel, Switzerland) and 1 mg/mL of DNase I in HBSS] using a 30 g needle, cut into small pieces, and placed into C-tubes (Miltenyi Biotec) containing 4 mL of digestion buffer. C-tubes were placed on a GentleMACS dissociator (Miltenyi Biotec) and run on the m_brain_3 protocol, after which they were placed in an incubator for 30 min at 37°C with shaking at 200 rpm. After incubation, C-tubes were placed back on the GentleMACS dissociator and run on the m_brain_3 protocol. Released cells were passed through a 40 μm nylon mesh using a cell masher and washed with 100 ml of wash buffer (autoMACS Running Buffer, Miltenyi Biotec). Astrocytes, endothelial cells, microglia, and infiltrating cells were isolated using a 30/70 percoll gradient (Percoll Plus, GE Healthcare). The cells collected from the gradient interphase were washed with HBSS and counted using an automated cell counter (Countess, Invitrogen); dead cells were discriminated using trypan blue. Cells were stained with live/dead Aqua viability dye (Invitrogen), incubated with Fc-block (BD Bioscience), and stained with fluorochrome-conjugated antibodies (listed in Supplementary Table S1). Data were acquired on a BD FACSAria cell sorter (BD Biosciences, San Jose, CA). Infiltrating T cells (CD45^high^ CD4^+^ CD8^+^), B cells (CD45^high^ B220^+^ MHC II^+^), macrophages (CD45^high^ CD11b^+^ CD64^+^) and eosinophils (CD45^high^ CD11b^+^ SiglecF^+^ SSC^high^) were quantified, and astrocytes (CD45^−^ GFAP^+^), endothelial cells (CD45^−^ GFAP^−^ CD31^+^) and microglia (CD45^low^ CD11b^+^ CD64^+^) were sorted for RNA sequencing and downstream analysis. Pelleted sorted cells were immediately lysed in extraction buffer from a PicoPure RNA isolation kit (Arcturus Bioscience), and lysates were stored at −80°C until RNA was extracted. Analysis of the flow cytometric data was performed using Flowjo software (TreeStar, Ashland, OR).

### RNA sequencing (RNA-seq)

RNA from FACSorted astrocytes, endothelial cells, and microglia of age-matched fingolimod treated and control mice were extracted using a PicoPure RNA isolation kit according to manufacturer's instructions. Sample quality control, processing, and library preparation were performed by the Northwestern University Next Generation Sequencing Core. RNA quality and quantity were measured using Agilent High Sensitivity RNA ScreenTape System (Agilent Technologies). RNA-seq libraries were prepared from 3 ng of total RNA using the QuantSeq 3′ biased mRNA-Seq Library Prep Kit for Illumina (Lexogen). DNA libraries were sequenced on an Illumina NextSeq 500 instrument with a target read depth of ~10 million reads per sample.

### RNA-seq analysis

Raw sequencing files were first de-multiplexed using bcl2fastq. The resulting fastq files were trimmed of low-quality reads and bases, polyA tails, and adaptors using bbduk (http://jgi.doe.gov/data-and-tools/bb-tools/). The trimmed fastq files were aligned to the mouse reference genome (mm10, Genome Reference Consortium GRCm38) using the STAR (Spliced Transcripts Alignment to a Reference) algorithm (39). HTSeq was run on the resulting BAM files to provide raw gene counts. Raw gene counts for each sample were merged into a single gene expression table and normalized for read depth using counts per million (CPM). For astrocytes, four and three of the highest quality samples from control and treated groups, respectively, were included for subsequent analyses. One sample was eliminated to due contamination with primer dimers in the library preparation, and two samples were eliminated due to low gene counts at a given expression level, one of which also had the lowest % uniquely mapped reads within its group. Astrocyte sample libraries showed an average of 83% alignment to the reference genome. For endothelial cells, four of the highest quality samples from control and treated groups were included for subsequent analyses. Two samples were eliminated due to low gene counts at a given expression level, both of which had the lowest % uniquely mapped reads within their experimental groups. Endothelial cell sample libraries showed an average of 80% alignment to the reference genome. For microglia, four of the highest quality samples from the treated group and five samples from the control group were included for subsequent analyses. One sample was eliminated to due contamination with primer dimers in the library preparation. Microglia sample libraries showed an average of 81% alignment to the reference genome. For all cell types, differential expression analysis was performed using DESeq2 (version 1.10.1) (40) and R (version 3.4.2). Briefly, expression count matrices were fit to a generalized linear model per gene following a negative binomial distribution. Dispersion estimates for each gene within groups were shrunk using an empirical Bayesian approach using default DESeq2 parameters. Log_2_ fold changes were compared between disease groups using the Wald test. Pathway enrichment analyses were performed by enrichR using the Reactome 2018 (41, 42) and Gene Ontology Consortium Biological Processes using genes with a *p*-value of < 0.05 and fold change in expression between experimental groups >1. Genes with 0 expression across all samples were excluded.

### Multiplex cytokine array

Protein was extracted from previously snap-frozen cortex and hippocampus homogenates from treated and control mice using T-PER tissue protein extraction buffer (ThermoFisher Scientific) following centrifugation. Protein concentrations were normalized and analyzed using the LEGENDPlex Mouse Inflammation Panel (Biolegend, San Diego, CA), a flow-cytometry based assay. Cytokines assessed include IL-1α, IL-1β, IL-6, IL-10, IL-12p70, IL-17A, IL-23, IL-27, MCP-1, IFN-β, IFN-γ, TNF-α, and GM-CSF.

### Statistical analysis

All statistical analysis was performed using GraphPad Prism software (La Jolla, CA) and JMP12 (SAS, Cary, NC). Normality was determined with the D'Agostino and Pearson normality test and, for most experiments, significant effects between groups of mice were determined by two-tailed Student's *t*-test or Mann-Whitney U-test. Since the effects of treatment in the two independent experimental cohorts were similar, fingolimod and control treated mice from both cohorts were respectively combined for the analysis. For OP and OR tasks, the threshold for preference was set at >55 %. “Preference” vs. “no preference” was then analyzed by chi-square and Fisher's exact test between groups. Results are displayed as the mean ± SEM of control versus treated. For the immunofluorescent and immunohistochemical staining studies, absolute cell number or mean fluorescent intensity was quantified when calculating the differences between the groups. When for technical reasons not all mice were studied in several of the individual assays, the subset of mice to be studied was chosen randomly. For all analyses, significance was defined as *p* < 0.05.

## Results

### Fingolimod treatment reduces depression-like behavior in MRL/lpr mice

MRL/lpr mice exhibit disturbed emotionality as early as 6 weeks of age (31) and cognitive impairment as early as 10 weeks of age (43). We therefore started fingolimod treatment at 10 weeks of age to ensure that the underlying neuropsychopathology would already be established in the study cohort. To determine the effects of fingolimod treatment on the development of neuropsychiatric disease in the MRL/lpr mouse, we utilized an extensively validated battery of neurocognitive and behavioral assessments after 4 weeks of treatment. MRL/MpJ (MRL/+) mice do not develop CNS disease (27), and they were therefore not included in the study.

**Figure 1:**
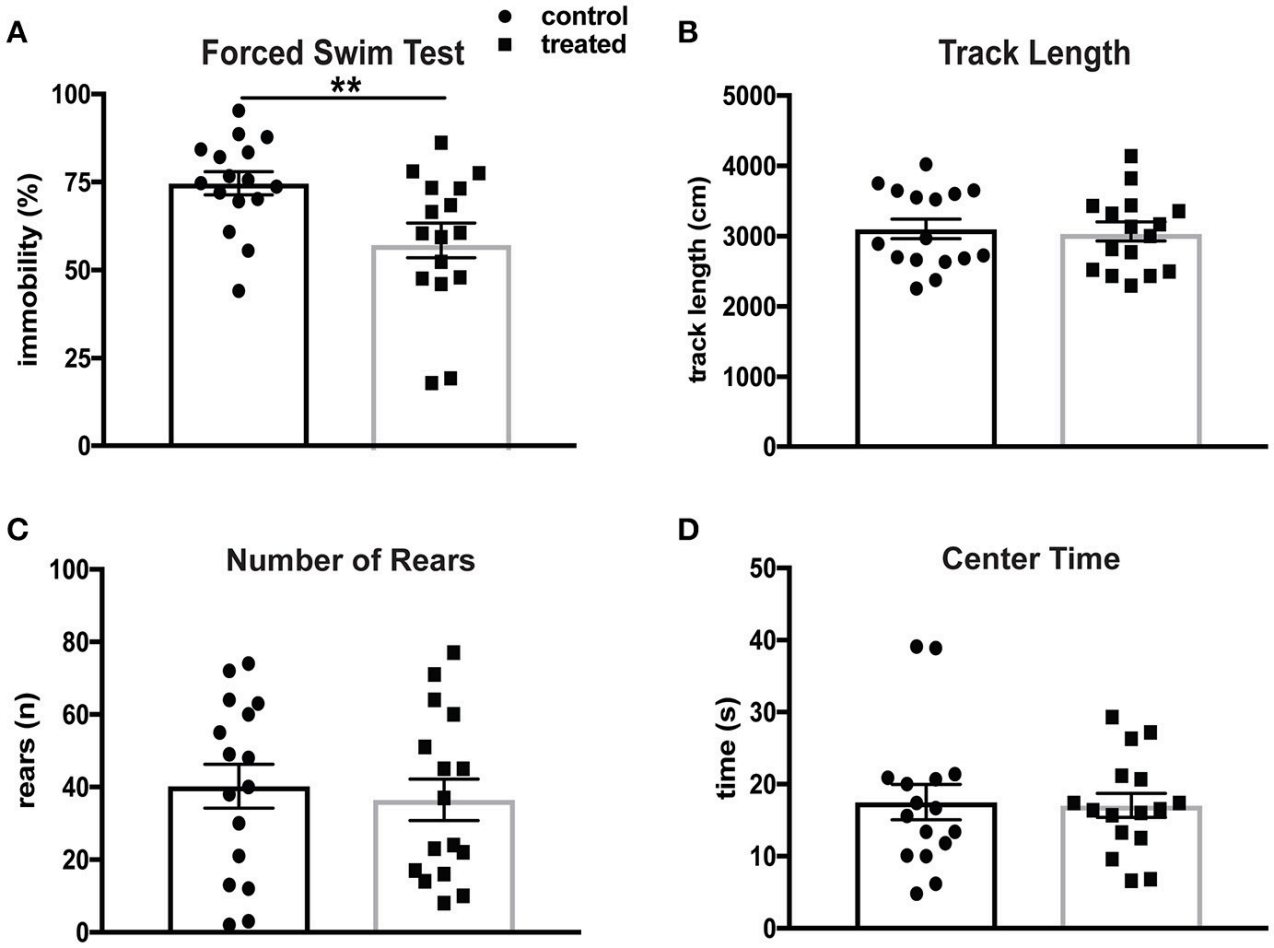
Fingolimod treatment mitigates depression-like behavior in lupus prone MRL/lpr mice. **(A)** Despair-induced immobility in treated and control mice during the last 3 minutes of the forced swim test was measured by time spent immobile and reported as a percent of total time. **(B–D)** General locomotor activity was assessed in both groups in the behavioral spectrometer and recorded by Viewer software. The total track length **(B)**, number of rears **(C)**, and amount of time spent in the 15 × 15 cm center area **(D)** were assessed. Control treated, *n* = 16; Fingolimod treated, *n* = 16. ^**^p < 0.01. Results are displayed as mean ± SEM.

Depression is the most common affective disorder in NPSLE patients, and the prevalence of mood disorders has been estimated to be 65% (44). The Porsolt forced swim test (FST) assesses depression-like behavior based on time spent immobile in a beaker of room temperature water. This test is based on the principle that mice without emotional deficits swim and/or struggle when immersed in water. MRL/lpr mice have been shown to exhibit significantly increased immobility in the FST at 6 weeks of age (31). Mice treated with fingolimod (*n* = 16) and controls (*n* = 16) were evaluated by the FST after 4 weeks of treatment at 14 weeks of age. Fingolimod treatment significantly reduced the total amount of time spent immobile. Figure 1A shows the difference in immobility during the last 3 min of the test (control vs. treated: 76.4 vs. 58.5%, *t*_(30)_ = 2.76, *p* = 0.0098). The behavioral spectrometer, which measures general locomotor activity and other behaviors, was used to determine if fingolimod treatment affected activity, exploration, and anxiety-like behavior. There were no significant differences in total track length (control vs. treated: 3,037 ± 134 cm vs. 3,104 ± 140 cm, *t*_(30)_ = 0.34, *p* = 0.73, Figure 1B), number of rears (control vs. treated: 36.5 ± 5.2 vs. 40.25 ± 6.1, *t*_(30)_ = 0.45, *p* = 0.66, Figure 1C), and amount of time spent in the center (control vs. treated: 16.6 ± 1.9 sec vs. 16.2 ± 1.5 sec, U = 123, *p* = 0.85, Figure 1D) between fingolimod and control treated mice. Moreover, the time spent running, orienting, and remaining still were also not different between the two groups (data not shown). This suggests that the differences seen in the FST after fingolimod treatment are not the result of alterations in locomotion, but rather are due to a reduction of the disturbed emotionality seen in MRL/lpr mice at this age.

### Visual and recognition memory impairments in MRL/lpr mice are attenuated with fingolimod treatment

**Figure 2:**
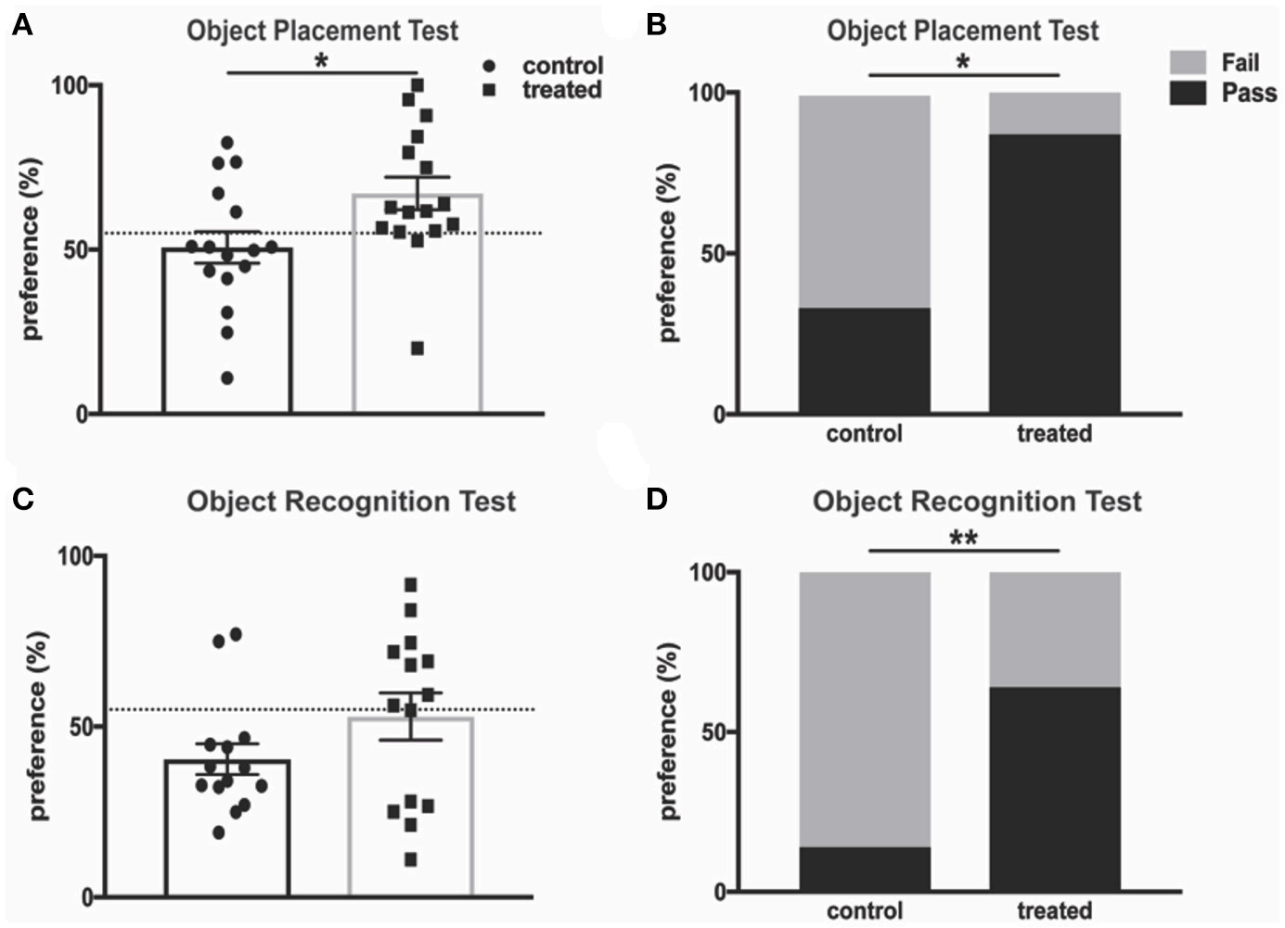
Fingolimod treatment improves spatial and recognition memory deficits. **(A,B)** Spatial and **(C,D)** recognition memory were assessed with the **(A,B)** object placement test and **(C,D)** object recognition test. **(A)** The preference score of each mouse is reported as percent of total exploration time that is spent exploring **(A)** the object in a novel position and **(C)** the novel object. The dotted line denotes 55% preference, and a preference score above is considered “passing.” The percent of mice in each group with a passing score is reported for the object placement test **(B)** and object recognition test **(D)**. Control treated *n* = 16; Fingolimod treated, *n* = 16. Two mice per group were excluded from the object recognition test for failure to explore for a minimum of 3 seconds during the training interval. ^*^p < 0.05, ^**^p < 0.01. Results are displayed as mean ± SEM.

Cognitive impairments, including memory deficits, are prevalent symptoms of neuropsychiatric disease in lupus patients and animal models of the disease, and these deficits have been extensively studied in MRL/lpr mice (43). Fingolimod-treated mice and controls were subjected to the object placement (OP) test, which evaluates spatial memory and is predicated on the tendency of mice to preferentially explore novel objects. MRL/lpr mice aged 10–20 weeks demonstrate reduced preference for a newly positioned object in this test when compared with MRL/+ controls (43). We found that 4 weeks of fingolimod treatment significantly increased novel (displaced) object preference when compared with PBS-injected controls (control vs. treated: 50.7% ± 4.8 vs. 67.1% ± 5.0, *t*_(30)_ = 2.37, *p* = 0.025, Figure 2A). Mice with a preference score above 55% are considered to have passed the test, and a significantly increased percentage of treated mice passed when compared with controls (33.3% vs. 87.5%, *p* = 0.003, Figure 2B). We also performed the object recognition (OR) test, which tests recognition memory when a novel object is introduced, after 4 weeks of treatment. Fingolimod treated mice tended to explore the novel object preferentially, with some reduction of recognition memory deficits when compared with controls (control vs. treated: 40.5% ± 4.5 vs. 53.0% ± 6.9, U = 76, *p* = 0.33, Figure 2C). Moreover, the percent of mice that passed was significantly increased in the fingolimod treated group (control vs. treated: 14.3 vs. 85.7%, *p* = 0.02, Figure 2D). Similar results were found when the OP and OR tests were repeated after 8 weeks of treatment (data not shown). Taken together, these results suggest that fingolimod has a positive and significant effect on treating cognitive deficits in MRL/lpr mice.

### Fingolimod reduces leukocyte infiltration of the choroid plexus

**Figure 3:**
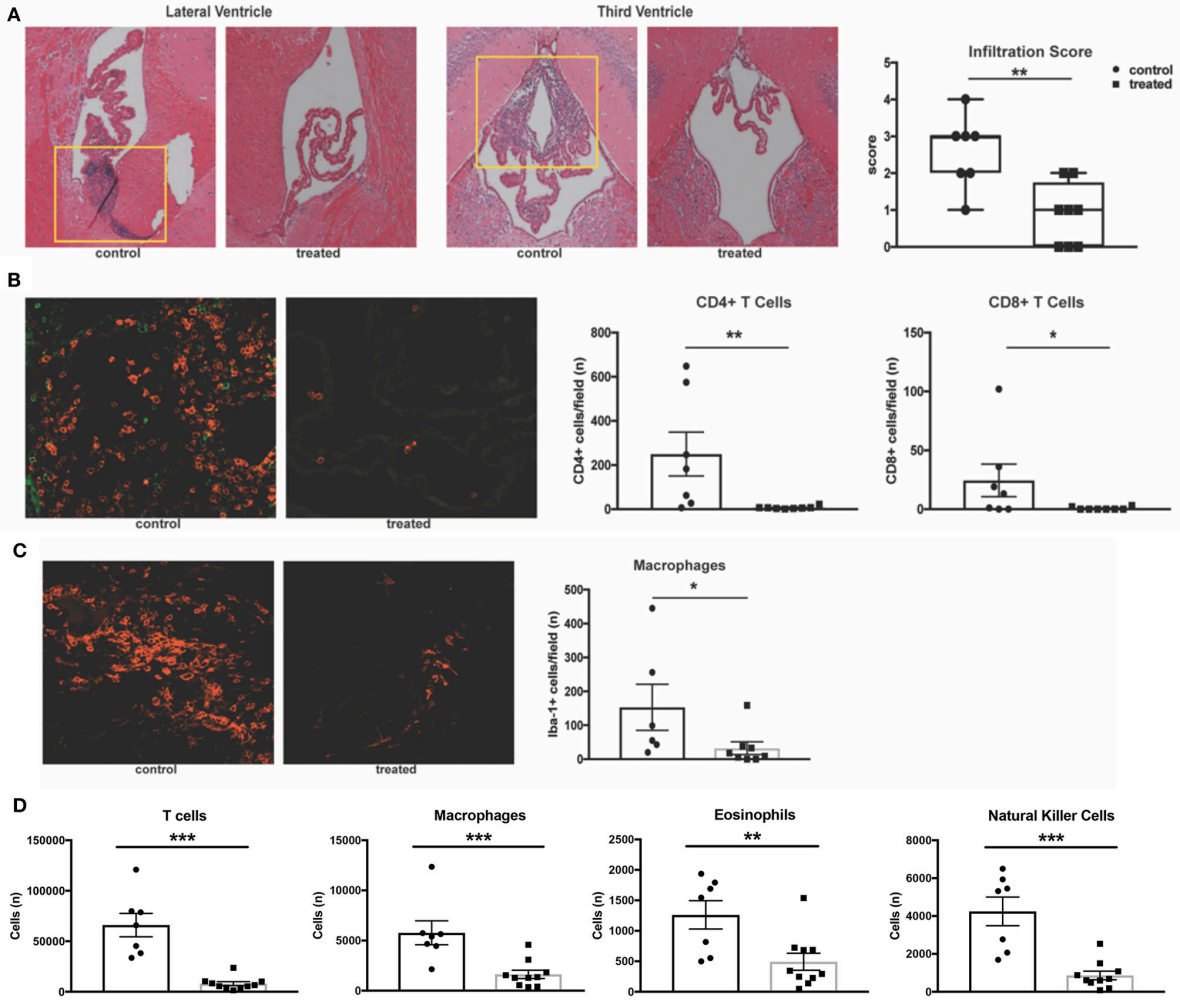
Fingolimod significantly reduces CNS infiltration. **(A)** Coronal sections of brains from fingolimod treated mice and controls were stained with H&E, and the choroid plexus was blindly scored for lymphocytic infiltration (10x magnification). Representative sections are shown. **(B)** Immunofluorescent staining of CD4+ T cells (magenta) and CD8+ T cells (green) and quantification of cells per field at 20x magnification. **(C)** Iba1+ macrophages (magenta) were also quantified at 20x magnification. Control treated, *n* = 7; Fingolimod treated, *n* = 8. **(D)** Quantification of infiltrating T cells, macrophages, eosinophils, and natural killer cells by flow cytometry from hemisected brain lysates. Control treated, *n* = 10; Fingolimod treated, *n* = 7. ^*^p < 0.05, ^**^p < 0.01, ^***^p < 0.001. Results are displayed as mean ± SEM.

Previous work has demonstrated that MRL/lpr mice exhibit severe neuroinflammation. The stroma of the choroid plexus becomes heavily infiltrated with T cells, B cells, and macrophages migrating from the periphery, and the extent of this infiltration correlates with disease severity (43). Given the proposed mechanism of action of fingolimod, we hypothesized that treated mice would exhibit less trafficking of lymphocytes into the central nervous system. H&E staining of coronal sections revealed a significant decrease in the incidence of cellular infiltration in the lateral ventricles of fingolimod treated mice (data not shown). Moreover, infiltration severity was significantly decreased (control vs. treated: median = 3 vs. 1, U = 5.5, *p* = 0.008, Figure 3A). To further characterize this infiltration, immunofluorescent staining was performed on coronal sections from treated and control mice. CD3 staining for T cells showed a significant reduction in the number of T cells per field in treated mice (data not shown). Given that the double negative (CD3+CD4-CD8-) T cell subset is expanded in the MRL/lpr strain, additional CD4 and CD8 staining was performed. We identified that CD4+ T cells were the predominant infiltrating lymphocyte, and that numbers of both CD4+ (control vs. treated: 249.6 ± 99.2 vs. 6.8 ± 2.3, U = 3.5, *p* = 0.0023) and CD8+ (control vs. treated: 24.4 ± 13.9 vs. 0.63 ± 0.4, U = 12, *p* = 0.049) T cells per field were significantly reduced in the lateral ventricles of fingolimod treated mice (Figure 3B). Additionally, Iba-1 staining revealed a significant decrease in the number of infiltrating macrophages (153 ± 67.8 vs. 32.9 ± 18.7, U = 6, *p* = 0.02, Figure 3C).

To confirm the immunofluorescent studies and ensure the findings were representative, flow cytometric quantification of infiltrating leukocytes in hemisected brain samples was performed. Analysis found a significant reduction in the number of T cells (control vs. treated: 66016 ± 11575 vs. 7908 ± 1971, U = 0, *p* = 0.0001, Figure 3D) and macrophages (control vs. treated: 5775 ± 1192 vs. 1629 ± 414, U = 3, *p* = 0.0007, Figure 3D) with fingolimod treatment. Additionally, fingolimod decreased the number of Ly6G+ myeloid cells (control vs. treated: 4329 ± 666 vs. 1606 ± 516, U = 8, *p* = 0.007, data not shown), eosinophils (control vs. treated:1262 ± 233 vs. 493± 139, U = 9, *p* = 0.0097, Figure 3D), and natural killer cells (control vs. treated: 4246 ± 756 vs. 864 ± 227, *t*_(15)_ = 4.97, *p* = 0.0002, Figure 3D) in the brain. There was no significant reduction of infiltrating B cells per field evident by immunofluorescence staining of B220+ cells in the lateral ventricle (control vs. treated: 65 ± 37 vs. 38 ± 37, U = 16.5, *p* = 0.19, data not shown) or by flow cytometry (control vs. treated: 2515 ± 651 vs. 1448 ± 421, U = 18, *p* = 0.11, data not shown). The reduction of leukocyte infiltration in MRL/lpr mice by fingolimod treatment demonstrates reversal of a main contributor to the damaging neuroinflammatory cascade that is believed to underlie NPSLE.

### Fingolimod has no effect on cortical and hippocampal IgG deposition in MRL/lpr mice

**Figure 4:**
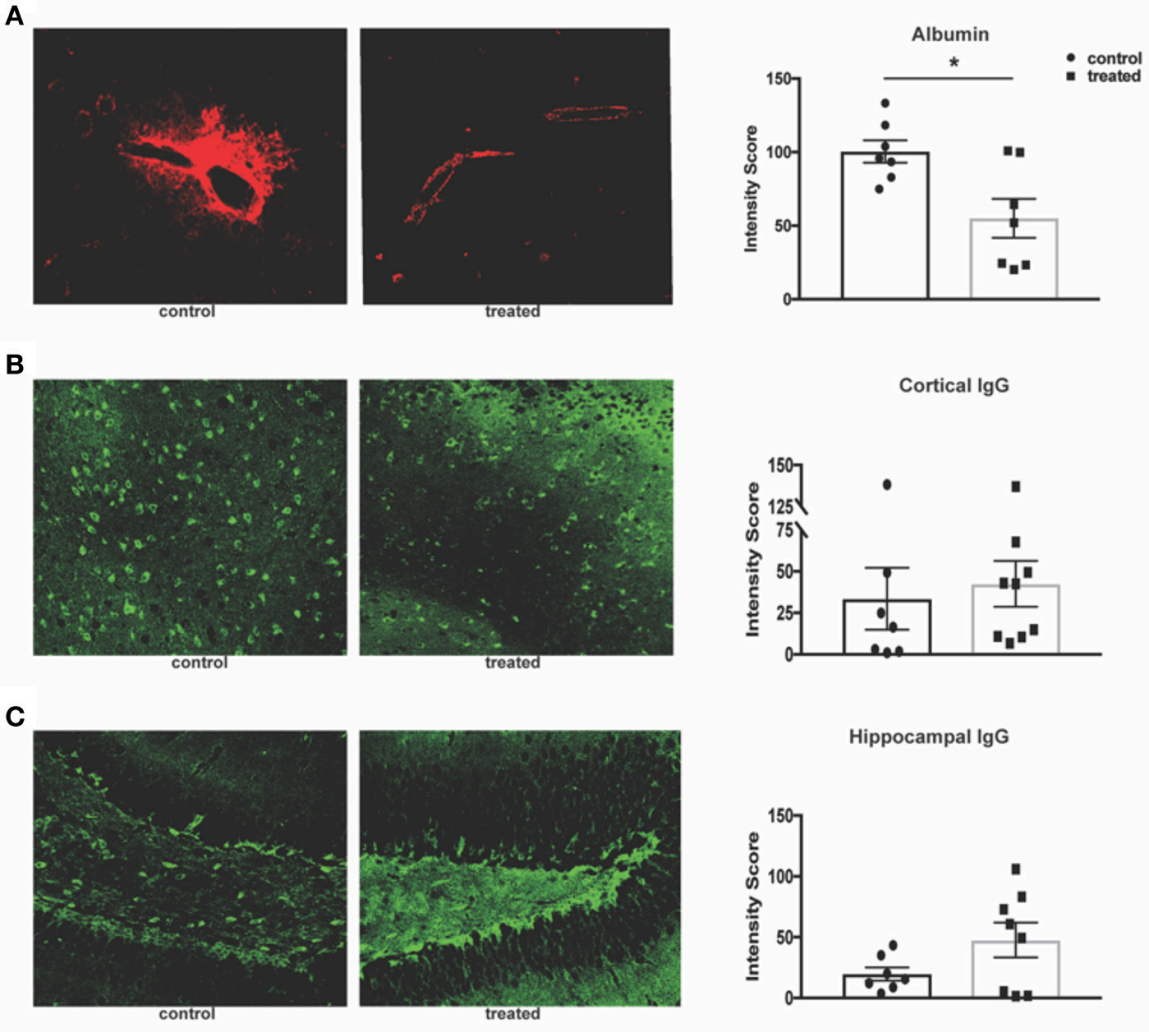
Fingolimod reduces albumin leakage but has no effect on IgG deposition. **(A)** Leakage of serum albumin (red) around cortical vessels was quantified by mean fluorescent intensity at 20x magnification **(B,C)**. Diffuse deposition of IgG (green) in the cortex **(B)** and hippocampus **(C)** was assessed by mean fluorescent intensity at 20x magnification to determine the intensity score. Representative sections are shown. Control treated, *n* = 7; Fingolimod treated, *n* = 8. ^*^p < 0.05. Results are displayed as mean ± SEM.

Given fingolimod's effect on leukocyte infiltration and its reported effects on maintenance of the blood brain barrier (BBB) integrity, we assessed the permeability of the endothelial barrier through quantification of serum albumin and IgG leakage into the brain parenchyma. Fingolimod-treated mice display significant reduction of albumin leakage around cortical vessels (control vs. treated: 100.4 ± 7.6 vs. 55.1 ± 15.2, *t*_(12)_ = 2.97, *p* = 0.012, Figure 4A). However, deposition of IgG within the cortex (control vs. treated: 33.5 ± 18.6 vs. 42.5 ± 13.8, U = 23, *p* = 0.41, Figure 4B) and dentate gyrus of the hippocampus (control vs. treated: 19.8 ± 5.5 vs. 47.6 ± 14.3, *t*_(13)_ = 1.72, *p* = 0.11, Figure 4C) was unchanged between treated and control mice. This suggests that the mechanism by which fingolimod improves cognition and affective behaviors may not be dependent on autoantibody deposition in the cortex and hippocampus.

### Fingolimod reduces expression of inflammation-associated genes in astrocytes and endothelial cells

**Figure 5:**
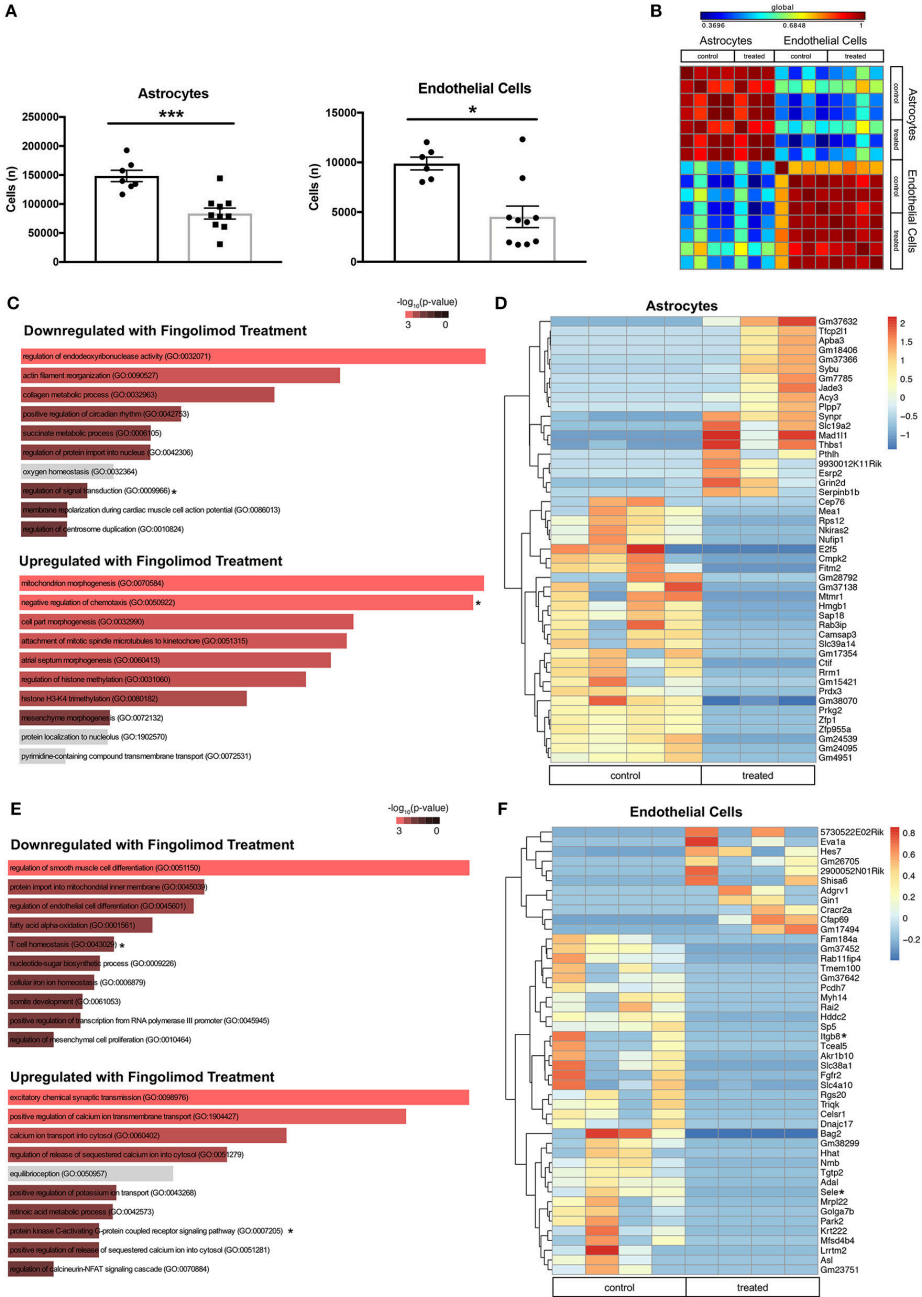
Fingolimod downregulates expression of inflammatory genes in astrocytes and endothelial cells. **(A)** Quantification of astrocytes (left) and endothelial cells (right) purified by fluorescence-activated cell sorting. ^*^p < 0.05, ^***^p < 0.001. Results are displayed as mean ± SEM. **(B)** Correlation matrix showing the differential expression of 12,000 genes demonstrates separation of astrocyte and endothelial cell samples. **(C,E)** Gene Ontology (GO) processes downregulated (top), and upregulated (bottom) in astrocytes **(C)** and endothelial cells **(E)** by fingolimod treatment. Significantly different processes are highlighted in red. Asterisks denote processes discussed in the text. **(D,F)** Heatmap of variably expressed genes that exhibited a significant difference (*p* < 0.05) between control and treated astrocytes **(D)** and endothelial cells **(F)** with a log2fold change of >6.5 and >5.5, respectively. Asterisks indicate genes discussed in the text. The color scale bar of the heatmaps reflects mean centered VST (Variance Stabilizing Transformation) units. **(C,D)** Control treated, *n* = 4; Fingolimod treated, *n* = 3. **(E,F)** Control treated, *n* = 4; Fingolimod treated, *n* = 4.

Astrocytes and endothelial cells, two major components of the BBB, are known targets of fingolimod's action (23, 45), and we hypothesized that treatment with the drug would modulate their activation. Flow cytometry performed on hemisected brain lysates revealed a significant decrease in the number of astrocytes (control vs. treated: 148429 ± 9734 vs. 83596 ± 9525, *t*_(15)_ = 4.62, *p* = 0.0003, Figure 5A) and endothelial cells (control vs. treated: 9885 ± 646 vs. 4528 ± 1079, U = 8, *p* = 0.016, Figure 5A) in fingolimod-treated mice, suggesting a reduction in reactive astrogliosis and alterations in vascularization. GFAP+ astrocytes and CD31+ endothelial cells from treated and untreated mice were purified by flow cytometry, and gene expression profiles were assessed. A correlation matrix of the 12000 genes captured from the samples revealed clear separation of two distinct populations (Figure 5B).

Gene Ontology (GO) terms associated with genes significantly up- or down regulated by fingolimod in astrocytes are shown in Figure 5C and Supplementary Table S2. Genes down regulated in treated mice are associated with processes involved in the immune response, including *regulation of interferon-gamma production* (*p* = 0.041), *myeloid cell differentiation* (*p* = 0.043), *T helper cell differentiation* (*p* = 0.018), *regulation of signal transduction* (*p* = 8.37 × 10^−4^), and *positive regulation of lymphocyte differentiation* (*p* = 0.034). GO terms associated with upregulated genes in astrocytes after fingolimod treatment include *positive regulation of TGF*β *receptor signaling pathway* (*p* = 0.017), *regulation of IL-12 production* (*p* = 0.046), *positive regulation of IL-1b* (*p* = 0.041), and *negative regulation of chemotaxis* (*p* = 0.006). 563 genes were differentially expressed between astrocytes from control and treated mice with a p value of < 0.05. Those with a log2 fold change of 6.5 or greater are shown in Figure 5D. In addition, several immune-related genes, including *Hmgb1* (*p* = 1.14 × 10^−4^), *Cd74* (*p* = 0.046), *Cd274/Pdl1* (*p* = 0.012), *Irgm1* (*p* = 0.019), and *Iigp1* (*p* = 9.32 × 10^−5^) were downregulated in treated astrocytes, while genes involved with inhibiting the immune response, such as *Nfkbib* (*p* = 0.0016) and *Tgif2* (*p* = 0.042) were upregulated (Supplementary Table S2).

In the sorted endothelial cells, similar GO terms were enriched among the genes significantly downregulated with fingolimod treatment, including *T cell homeostasis* (*p* = 0.013), *positive regulation of macrophage activation* (*p* = 0.034), and *positive regulation of leukocyte activation* (*p* = 0.031; Figure 5E and Supplementary Table S3). Pathways implicated in the pro-inflammatory response, including *regulation of Wnt signaling pathway* (*p* = 0.035) and *regulation of fibroblast growth factor receptor signaling pathway* (*p* = 0.046) were also downregulated. In genes upregulated with fingolimod treatment, processes related to cell signaling, including *cAMP-mediated signaling* (*p* = 0.0045) and *protein kinase C-activating G-protein coupled receptor signaling pathway* (*p* = 0.0056) were evident (Figure 5E and Supplementary Table S3). Analysis revealed 654 genes exhibiting significantly differential expression between control and fingolimod treated endothelial cells, and those with a log 2 fold change greater than 5.5 are shown in Figure 5F. Genes involved in cellular adhesion, including *Sele* (*p* = 3.54 × 10^−4^), which encodes E-selectin, and *Itgb8* (*p* = 0.014), which encodes integrin beta-8, and in the interferon response, including *Irgm1* (*p* = 3.76 × 10^−5^) and *Iigp1* (*p* = 2.53 × 10^−6^), were among those most significantly downregulated. Signaling genes upregulated by fingolimod include *Nfkbid* (*p* = 7.04 × 10^−3^), *Map4k4* (*p* = 0.018), and *Irf1* (*p* = 0.034; Figure 5F and Supplementary Table S3).

Taken together, transcriptome analysis of astrocytes and endothelial cells from treated mice revealed reduced responsiveness to pro-inflammatory signaling, and these changes likely contributed to stabilization of brain barrier function in fingolimod treated mice.

### Microglia express increased interferon-beta and modulate immune pathways upon fingolimod treatment

**Figure 6:**
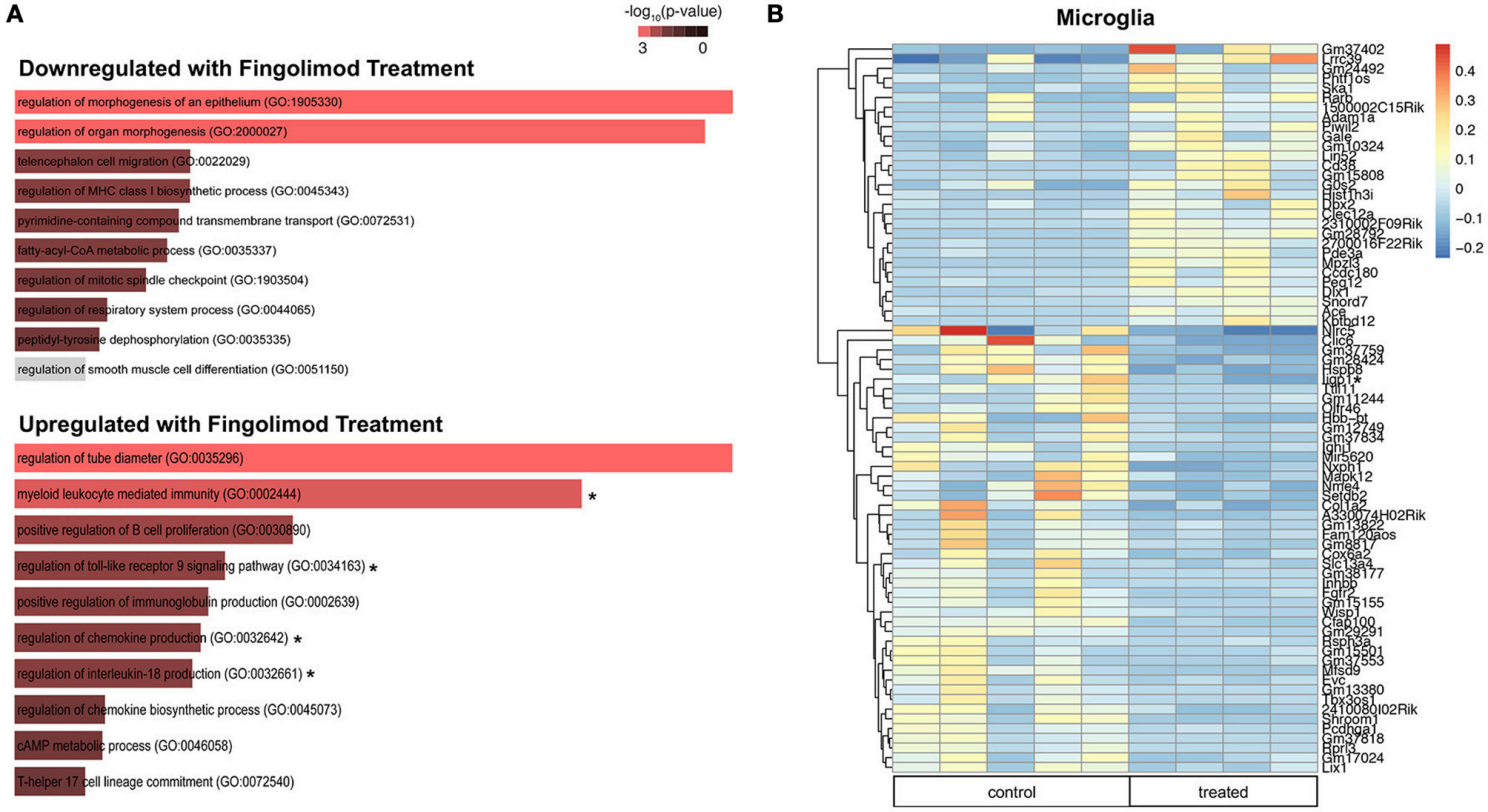
Fingolimod modulates key immune pathways in microglia. **(A)** Gene Ontology (GO) processes downregulated (top) and upregulated (bottom) in microglia by fingolimod treatment. Significantly different processes are highlighted in red. Asterisks denote processes discussed in the text. **(B)** Heatmap of variably expressed genes that exhibited a significant difference (*p* < 0.05) between control and treated microglia with a log2fold change of >2.5. The color scale bar of the heatmap reflects mean centered VST (Variance Stabilizing Transformation) units. Asterisks indicate genes discussed in the text. Control treated, *n* = 5; Fingolimod treated, *n* = 4.

Microglia are key regulators of immune homeostasis in the CNS, and fingolimod has been shown *in vitro* to reduce pro-inflammatory signaling and promote the release of neurotrophic factors (22). Several regulatory processes were downregulated in fingolimod treated mice, including *negative regulation of type I interferon-mediated signaling pathway* (*p* = 0.046) and *regulation of cell matrix adhesion* (*p* = 0.048; Figure 6A and Supplementary Table S4). Furthermore, we found that several GO processes and pathways related to the immune system were significantly upregulated in microglia sorted from fingolimod treated mice. Processes include *myeloid leukocyte mediated immunity* (*p* = 9.4 × 10^−4^), *regulation of toll-like receptor 9 (TLR9) signaling pathway* (*p* = 0.037), *regulation of IL-18 production* (*p* = 0.037), *regulation of chemokine production* (*p* = 0.011); pathways were *Map3/Erk1 activation* (*p* = 0.046), *Map1/Erk2 activation* (*p* = 0.041), and *IL-6 signaling* (*p* = 0.050; Figure 6A and Supplementary Table S4). Unsupervised clustering of the 50 most variably expressed genes revealed separation/clustering of treated and untreated samples, demonstrating a striking effect of treatment on microglia (data not shown). Of note, this clustering revealed that *Ifnb1* was highly upregulated in fingolimod-treated microglia. Genes with a log2fold change >2.5 that were significantly altered by fingolimod are shown in Figure 6B. Of the 665 genes that exhibited significant differential expression between control and untreated microglia, 335 were downregulated by fingolimod treatment. They include genes involved in NFkB signaling, such as *Nfkbid* (*p* = 0.030), *Nfkbib* (*p* = 0.018), and the interferon response, including *Ifrd1* (*p* = 5.82 × 10^−3^), *Ifi44* (*p* = 0.027), *Ifitm3* (*p* = 0.026), and *Iigp1* (*p* = 0.018) Upregulated genes of interest are involved in adhesion, such as *Itgam* (*p* = 5.34 × 10^−3^) and *Itgb2* (*p* = 7.9 × 10^−4^), the interferon response, such as *Isg20l2* (*p* = 0.037) and *Irf5* (*p* = 0.043), and pro-inflammatory signaling, including *Tlr9* (*p* = 0.031), *Il6* (*p* = 0.038), *Ccl12* (*p* = 0.038), and *Maf* (*p* = 0.024). (Figure 6B and Supplementary Table S4). These findings demonstrate that fingolimod modifies immune signaling in microglia, modulating several immunomodulatory cytokines and pathways and altering the interferon response.

### Cytokine protein expression in the cortex and hippocampus is not decreased by fingolimod treatment

**Figure 7:**
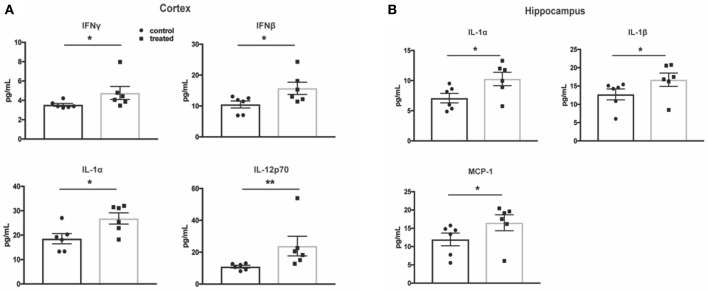
Fingolimod does not reduce pro-inflammatory cytokine expression in the cortex and hippocampus. **(A,B)** Cytokine protein levels were measured with a flow cytometry based multiplex cytokine assay using lysates from cortex **(A)** and hippocampus **(B)** samples. Control treated, *n* = 6; Fingolimod treated, *n* = 6. ^*^ *p* < 0.05, ^**^p < 0.01. Results are displayed as mean ± SEM.

One of the hallmarks of NPSLE is the presence of pro-inflammatory cytokines that perpetuate the neuroinflammatory cascade and result in parenchymal destruction (4). To assess fingolimod's effect on pro-inflammatory cytokine expression, a multiplex cytokine assay was used to quantify protein levels. Surprisingly, cortical samples from treated mice demonstrated higher levels of several pro-inflammatory cytokines, including IL-1α (control vs. treated:18.6 ± 2.1 pg/mL vs. 26.9 ± 2.3 pg/mL, *t*_(10)_ = 2.7, *p* = 0.02) and IL-12p70 (10.9 ± 0.8 vs. 23.9 ± 6.2, U = 1, *p* = 0.004, Figure 7A). The cortical samples also revealed increased levels of IFN-β (control vs. treated: 10.5 ± 1.1 pg/mL vs. 15.7 ± 2.0 pg/mL, *t*_(10)_ = 2.3, *p* = 0.04) and IFN-γ (control vs. treated: 3.5 ± 0.1 pg/mL vs. 4.8 ± 0.7 pg/mL, U = 4, *p* = 0.03), suggesting an interferogenic response to fingolimod (Figure 7A). Similarly, the hippocampus revealed increased levels of IL-1α (7.1 ± 0.8 pg/mL vs. 10.3 ± 1.1 pg/mL, *t*_(10)_ = 2.4, *p* = 0.04) as well as IL-1β (control vs. treated: 12.7 ± 1.5 pg/mL vs. 16.7 ± 1.8 pg/mL, U = 5, *p* = 0.04) and MCP-1 (control vs. treated: 12.0 ± 1.7 pg/mL vs. 16.5 ± 2.2 pg/mL, U = 5, *p* = 0.04; Figure 7B). The lack of a suppressive effect of fingolimod on cytokine levels highlights its immunomodulatory rather than immunosuppressive properties in the context of the chronic neuroinflammation evident in NPSLE.

### Fingolimod reduces organomegaly and circulating autoantibody titers

**Figure 8:**
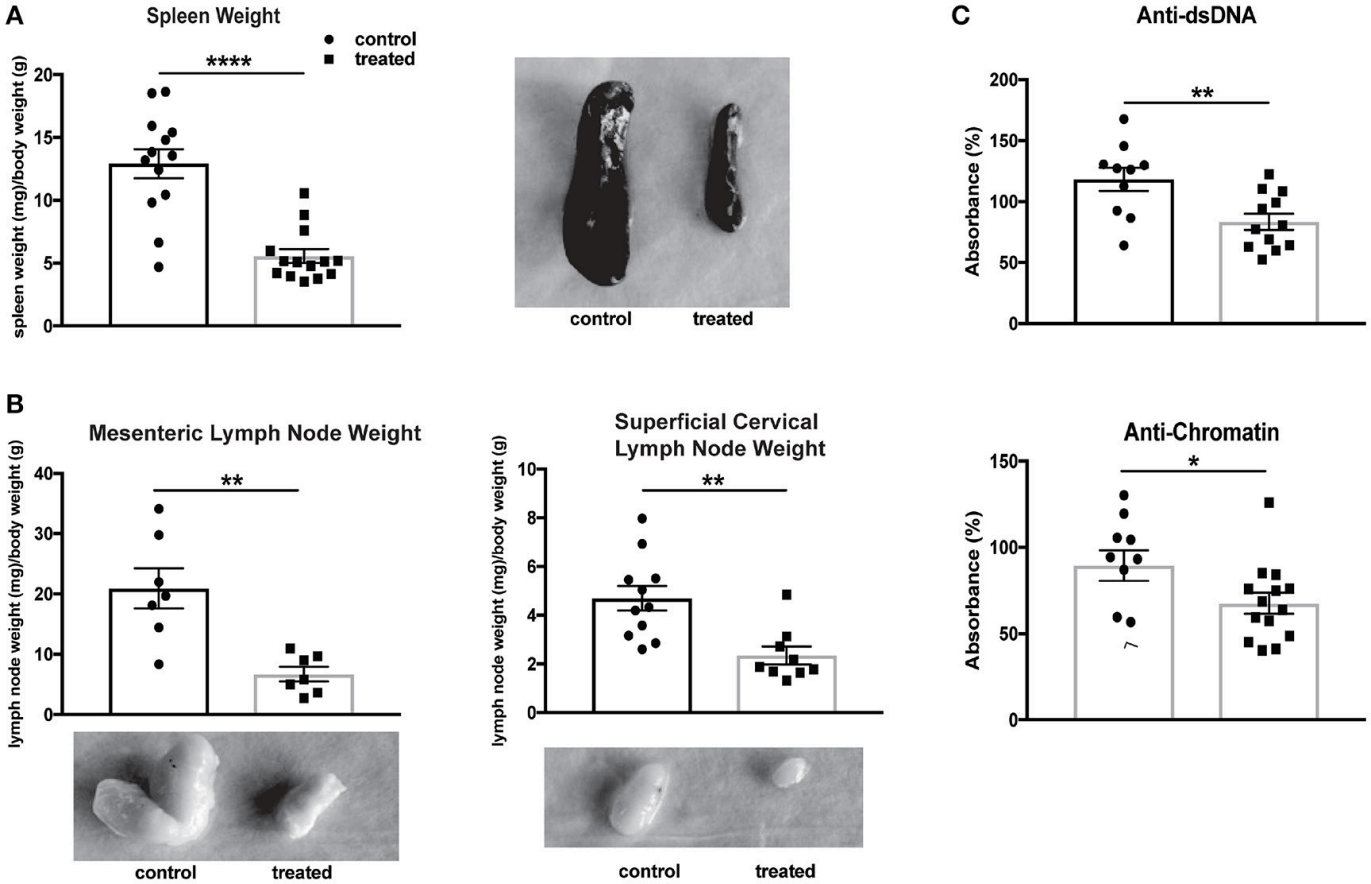
Fingolimod reduces splenomegaly, lymphadenopathy, and serum autoantibodies. **(A,B)** Spleens **(A)** and lymph nodes **(B)** from treated and control mice were weighed and corrected for body weight. Representative images of spleens **(A)** and lymph nodes **(B)** from control and treated mice are shown. Control treated, *n* = 7–16; Fingolimod treated, *n* = 7–16. **(C)** Antibodies to double stranded DNA and chromatin were assessed in serum from control and treated mice collected after 10 weeks of treatment (e.g., at 20 weeks of age). Data were normalized and are shown as a percentage of positive control. Control treated, *n* = 16; Fingolimod treated, *n* = 16. ^*^p < 0.05, ^**^p < 0.01, ^****^p < 0.0001. Results are displayed as mean ± SEM.

The systemic effects of fingolimod treatment on MRL/lpr mice have previously been studied, with reductions found in lymphadenopathy, splenomegaly, and anti-dsDNA antibodies (46). Fingolimod-treated mice in our study similarly demonstrated a significant diminution in spleen size (normalized to body weight; control vs. treated: 12.9 ± 1.2 vs. 5.6 ± 0.6, U = 14, *p* < 0.0001, Figure 8A) and in the size of regional lymph nodes, including mesenteric (control vs. treated: 20.9 mg ± 3.3 vs. 6.7 mg ± 1.2, *t*_(12)_ = 4.02, *p* = 0.002) and superficial cervical (control vs. treated: 4.7 ± 0.5 vs. 2.4 ± 0.4, U = 9, *p* = 0.001, Figure 8B) nodes. Finally, a reduction in anti-dsDNA antibody (control vs. treated: 118.3% ± 9.6 vs. 83.4% ± 6.6, *t*_(20)_ = 3.08, *p* = 0.006) and anti-chromatin (control vs. treated: 89.5% ± 8.9 vs. 67.6% ± 6.1, U = 36, *p* = 0.048) antibody titers was associated with fingolimod treatment (Figure 8C).

## Discussion

Neuropsychiatric manifestations of SLE significantly reduce the quality of life of SLE patients, and current treatment options are suboptimal (47). Here, we show that fingolimod, an FDA-approved treatment for relapsing-remitting MS, significantly ameliorated spatial and recognition memory deficits in lupus-prone MRL/lpr mice. These findings are in line with fingolimod's ability to counteract memory impairment in a dysbindin-1-deficient genetic model of cognitive deficits (48), a rat model of Huntington's disease (49), and a mouse model of Alzheimer's disease (50). The efficacy of fingolimod treatment in several disorders underscores the detrimental role of S1P signaling in neurocognitive impairment.

We also demonstrated attenuation of depression-like behavior in fingolimod-treated mice; this reduction in despair-induced immobility was not due to any differences in general locomotor activity. Similarly, MRL/lpr mice treated with cyclophosphamide, a cytotoxic and immunosuppressant drug, demonstrate increased sensitivity to sucrose, indicative of diminished anhedonia (51). It is intriguing that the depressive phenotype present in the MRL/lpr lupus strain is attenuated through an immunomodulating therapy. Depression however is also a feature of neurological disorders that do not have a prominent inflammatory or autoimmune basis (e.g., epilepsy), which would not be expected to respond to an immune response targeting therapy. Examining the immunological basis of affective disorders is a critical area of research, and the inflammatory hypothesis of depression expands upon the historical monoamine theory. Imbalance of biogenic amine neurotransmitters, such as norepinephrine, dopamine, and serotonin, in the limbic system and cortex have long been known to contribute to the pathogenesis of depression (52), and many antidepressants are aimed at increasing their bioavailability. Additionally, physiological and psychosocial stressors activate the sympathetic nervous system and hypothalamic-pituitary axis, resulting in increased NFkB signaling and pro-inflammatory cytokine production. These cytokines can modulate the release of neurotransmitters, contribute to excitotoxicity, and promote inflammatory responses in microglia and astrocytes (53). Indeed, antidepressants result in cytokine modulation (54), and fingolimod treatment may reduce depression through its effects on cytokine modulation and in reducing cellular infiltration, or interrupting the detrimental neuroinflammatory cascade that predicates depression. Furthermore, the genetic and epigenetic alterations that are associated with neuroinflammation in depression have yet to be fully elucidated. In addition to modulating inflammatory processes, fingolimod also inhibits histone deacetylases (HDACs) (55), an epigenetic modification associated with reduced depression (56). Interestingly, di Nuzzo et al. demonstrated the antidepressant activity of fingolimod in a chronic unpredictable stress model (57), highlighting the potential relevance of fingolimod in the treatment of depression outside of the autoimmune context as well. Future studies will be aimed at examining the effect of fingolimod on neurotransmitter availability and further pinpointing the mechanistic basis for its antidepressant effects in NPSLE.

Though diffuse NPSLE has a complex and multifactorial etiology, the major pathogenic factors are purported to be: (1) loss of BBB integrity, (2) the entry of autoantibodies into the CNS, and (3) upregulation of pro-inflammatory cytokines that result in a propagation of the neuroinflammatory milieu (4). Recent advances in our understanding of the neuroimmune interface and the neurovascular unit suggest that our assessment of CNS vasculature has been somewhat exclusive in its emphasis on the BBB; contributions of dysfunction of the other brain barriers, including the blood-cerebrospinal fluid barrier (BCSFB) and the meningeal barrier, to NPSLE pathogenesis demands further investigation (58). The choroid plexus, which is the principal component of the BCSFB, is a known site of severe lymphocytic infiltration in the MRL/lpr mouse model of lupus (43) and has also been implicated in SLE patients (59).

Histological examination of brain tissue demonstrated significant reduction in leukocyte infiltration of the choroid plexus in treated mice, particularly of T cells and macrophages. Flow cytometry confirmed these findings, and similarly demonstrated a significant reduction in the number of eosinophils and natural killer cells in the brains of fingolimod treated mice. The ability of fingolimod to reduce lymphocyte trafficking has been reported in MS models (11, 20), and this ability to reduce infiltration has also been shown to be beneficial in a model of hypoxic-ischemic injury (60). Considering the association between the severity of neuropsychiatric disease and brain lymphocytic infiltration, this specific effect of fingolimod provides additional support for its consideration as a potential therapeutic for NPSLE. Furthermore, since fingolimod is known to protect rodents from developing clinical manifestations of EAE when given early in the course of the disease (15), future studies will be aimed at investigating fingolimod's ability to fully prevent NPSLE development when administered in younger mice. It is however important to note that our results differ in several aspects from the reported effects and mechanism of action of fingolimod in MS models. This indicates that it is the immunomodulatory effects of fingolimod, rather than (or in addition to) its immunosuppressive properties, which may be important in the context of the neuroinflammation present in NPSLE.

Inflammatory activation of the cells that comprise the brain's barriers not only contribute to the immune response but also result in increased vascular permeability. Fingolimod has been shown to directly modify BBB composition through upregulation of tight junction proteins and adhesion molecule expression on endothelial cells (23); improvement of barrier integrity can reduce infiltration of peripheral mediators into the brain. In our study, fingolimod treated mice exhibited reduced extravasation of serum albumin in the perivascular brain parenchyma, which suggests a reduction of the permeability that is detrimental in NPSLE.

A multitude of factors, including endothelial cell activation, infection and stress, can result in a loss of barrier integrity that allows systemic factors such as autoreactive antibodies to enter the typically immunoprivileged CNS (61). Several antibodies, such as anti-ribosomal P (62) and anti-NMDA receptor (63) antibodies, have been positively correlated with NPSLE manifestations, and evidence of their pathological effects has been extensively reported (64). However, IgG deposition in the brain was unchanged, and the potential contribution of these autoantibodies to pathogenesis was modulated as is evident from fingolimod's beneficial effects on the neurobehavioral profile.

In addition to the well-characterized effect on lymphocytes, fingolimod readily crosses the BBB (17) and acts directly upon several cell types within the CNS that express S1P receptors (20). Previous studies have shown that astrocytes, which are key components of the neurovascular unit and perform diverse immunomodulatory functions, exhibit reduced pro-inflammatory cytokine production (65) and less reactive astrogliosis (66) after fingolimod treatment. Microglia, the resident immunocyte of the CNS, demonstrate reduced activation in a mouse model of MS (21, 67) and a neuroprotective M2 phenotype *in vitro* (22) after fingolimod treatment. Furthermore, hippocampal gliosis has previously been demonstrated in MRL/lpr mice in response to CNS injury and chronic inflammation (68).

In this study, we report for the first time a dissection of the cell-type specific effects of fingolimod on astrocytes, endothelial cells, and microglia in the context of chronic neuroinflammation. Transcriptional profiling revealed several GO terms and genes associated with inflammation and immune signaling that were significantly downregulated in astrocytes and endothelial cells from mice treated with fingolimod, in congruence with fingolimod's documented immunosuppressive effects. Interestingly, *Irgm1*, which encodes immunity-related GTPase family M member 1, was significantly downregulated by fingolimod in both astrocytes and endothelial cells. This interferon-inducible gene is upregulated in EAE and promotes BBB and blood-CSF barrier disruption (69). These findings suggest an immunoprotective role for fingolimod in the chronic inflammation seen in the MRL/lpr NPSLE model. In microglia, fingolimod treatment was associated with enhanced cellular adhesion, cytokine production (including IL-6, IL-8, and CCL12), and TLR9 signaling. Moreover, regulatory processes, including negative regulation of type-I interferon and interferon-stimulated genes were modulated. The type I interferon signature in SLE is complex (70) and has been extensively studied in neuroinflammation (71–73). IFN-beta 1a is used in the treatment of relapsing-remitting MS; interestingly, it also exhibits an anti-inflammatory role in focal cerebral ischemia (74). Moreover, *in vitro* experiments have demonstrated IFN-beta's neuroprotective role in response to activated microglia (75). The pleotropic and differential effects of fingolimod on various cell types, including some not yet studied, deserve additional attention in dedicated future studies.

Fingolimod has previously been investigated as a potential therapeutic for systemic lupus manifestations in several mouse models (46, 76, 77), but its mechanism of action in this disease was not previously explored in detail. In the MRL/lpr mouse, fingolimod reduced autoreactive antibodies, splenomegaly, lymphadenopathy, and immune complex deposition in the kidney (46). In the NZB/W F1 mouse, however, no change in anti-DNA autoantibodies or glomerular mesangial expansion was seen, and improvements were limited to reduction of proteinuria and immune complex deposition (76). Autoantibodies were again unchanged when fingolimod was administered to BXSB mice, but mesangial cell proliferation and glomerular infiltration were reduced (77). Although fingolimod exerted beneficial effects in all studies, variability in responses highlight the complex etiology of SLE. Moreover, the effects on neuropsychiatric disease were not assessed in these models. The reduction in organomegaly in the MRL/lpr mice is thought to be due to the propensity of fingolimod to induce apoptosis in the double negative T cell populations that are classically found in the MRL/lpr mice (46). However, double negative T cells are rare to absent in the brains of MRL/lpr mice (78). Fingolimod treated mice in our study also exhibited a difference in serum autoantibody titer, but IgG deposition in the CNS was unchanged (79). Nevertheless, it is possible that a general reduction in disease severity (e.g., attenuated systemic inflammatory milieu) also contributed to the improvements seen in our fingolimod treated mice.

Recently, fingolimod was shown to ameliorate behavioral deficits in B6.MRL/lpr mice (80), a model which had not previously been shown to exhibit a robust neuropsychiatric phenotype. In this cohort of only 4–6 mice per group and after 12 weeks of treatment, Shi et al. used the tail suspension test to measure depression-like behavior. However, the tail suspension test is not recommended for use in mice on a C57BL/6 background, which have a tendency to climb up their tails and skew latency assessment (81). Further, Shi et al. report that fingolimod treatment significantly decreased center track length and time spent in in the center during the open field test. This would indicate increased thigmotaxis and anxiety rather than an anxiolytic effect. In the present study, we observed significant amelioration of marked cognitive and affective deficits in a large cohort of MRL/lpr mice with severe disease after only 4 weeks of treatment using an established battery of tests, thereby providing a more robust model in which to examine therapeutic potential.

Several cytokines and chemokines, including IL-2 (82), IL-6 (83), IFNα (84), IFNγ (84), and TWEAK (85), are upregulated in the CSF of SLE patients, and many of these immune mediators have been investigated as potential biomarkers (86). These cytokines can be produced by infiltrating leukocytes or CNS resident cells such as glia and neurons in response to autoantibodies and other immune factors (47). Recently, microglia were shown to be stimulated by type I interferon to engulf neuronal material and prune synapses in a lupus model (87). The interplay between pro-inflammatory cytokines, glia, and neurons, their role in perpetuation of the neuroinflammatory cascade, and their contribution to the behavioral manifestations seen in NPSLE are still yet to be fully understood. Surprisingly, despite improvements in emotionality, cognition, and albumin leakage, fingolimod treatment did not reduce several pro-inflammatory cytokines in the hippocampus and cortex. Moreover, several cytokines, including IL-1α, IL-1β, and IFN-γ were increased with fingolimod treatment. This is in contrast with several studies that highlight a reduction in pro-inflammatory mediators as a central aspect of this drug's mechanism of action. Nevertheless, the increased IFN-γ found here may have had beneficial effects on immune cell trafficking and repair of brain injury mediated via the choroid plexus (88). Some studies have shown a lack of cellular responsiveness to cytokine challenge after fingolimod treatment (89), though this mechanism is not well understood. Given that S1P receptors exist on all of the major cell types involved in neuroinflammation, including astrocytes, endothelial cells, neurons, and microglia, it stands to reason that their lack of responsiveness to pro-inflammatory stimuli could be a possible pathway by which the inflammatory cascade is disrupted. Nevertheless, Yang et al. reported a reduction in lymphocytic infiltration with fingolimod treatment in a hypoxic-ischemic mouse model, without fingolimod directly inhibiting microglial activation *in vivo* and *in vitro* (60). This provides further evidence that fingolimod is capable of conferring a neuroprotective phenotype even in the absence of expected responses in brain resident cells.

In addition to its effects on neuroinflammation, fingolimod has also been shown to have direct effect on neurons through promoting hippocampal neurogenesis (90) and inhibiting excitotoxic neuronal apoptosis (25). Neuronal changes are an area of interest in NPSLE, with a recent study in a murine lupus model showing that synaptic pruning was dependent on the microglial response to interferon signaling (87). However, no apparent differences between fingolimod-treated and control mice in neuronal apoptosis was seen by terminal deoxynucleotidyl transferase dUTP nick-end labeling (TUNEL) staining (data not shown). This study also only explored the effects in the MRL/lpr mouse, and a comparison of effects in other mouse models of NPSLE may shed additional light on the pathogenic mechanisms that are altered with fingolimod treatment.

In summary, fingolimod significantly attenuated depression-like behavior and spatial and recognition memory deficits in lupus prone MRL/lpr mice, likely mediated through significantly reducing infiltration of T cells and macrophages in the choroid plexus. Systemically, treatment resulted in significant reduction of splenomegaly, lymphadenopathy, and circulating autoantibodies, and this reduction in disease burden could also contribute to the cognitive and affective improvements observed. Surprisingly, attenuation of the neurobehavioral deficits was not associated with a global reduction in brain cytokine levels; rather, the significant change to the neuroinflammatory milieu was via (1) reduction of brain leukocyte infiltration, (2) modulation of microglia signaling, including increased cytokine signaling and Erk1/Erk2 pathway activation, and (3) reduced pro-inflammatory and, specifically, interferon signaling in astrocytes and endothelial cells. These immunoregulatory changes are apparently a major contributor to the genesis of the neuropsychiatric phenotype in the MRL/lpr lupus strain. In conjunction with its already established use, these findings point to fingolimod as a promising but as yet unexplored therapeutic target in human lupus with involvement of the CNS.

## Author contributions

EM, AS, MG, CC, and CP conceived and designed the experiments. EM, HM, and CC performed the reported studies. GG and DW processed the transcriptional data. EM, HM, ED, MG, CC, and CP analyzed the data. EM, HM, AS, ED, MG, GG, DW, CC, and CP wrote and/or edited the paper, and approved the final submitted version.

### Conflict of interest statement

The authors declare that the research was conducted in the absence of any commercial or financial relationships that could be construed as a potential conflict of interest.

## Abbreviations

SLE: systemic lupus erythematosus
NPSLE: neuropsychiatric systemic lupus erythematosus
BBB: blood-brain barrier
BCSFB: blood-cerebrospinal fluid barrier
CNS: central nervous system
S1P: sphingosine-1-phosphate
ds: double stranded
MS: multiple sclerosis
TUNEL: terminal deoxynucleotidyl transferase dUTP nick-end labeling
GO: Gene Ontology
EAE: experimental allergic encephalomyelitis
H&E: hematoxylin and eosin
FST: forced swim test
OP: object placement
OR: object recognition.
