# Supplementary material for: Neuropsychiatric Systemic Lupus Erythematosus Is Dependent on Sphingosine-1-Phosphate Signaling
Source: Front Immunol. 2018 Sep 26;9:2189. doi: 10.3389/fimmu.2018.02189 (PMC6168636; doi:10.3389/fimmu.2018.02189)
Supplement: Supplementary file 1 [file Data_Sheet_1.docx]

**Supplementary Table S1.** List of antibodies utilized for brain flow cytometric analysis.

| **Antigen** | **Clone** | **Fluorochrome** | **Manufacturer** |
| --- | --- | --- | --- |
| CD45 | 30-F11 | FITC | eBioscience |
| CD64 | X54-5/7.1 | PE | Biolegend |
| CD31 | MEC 13.3 | PerCP-Cy5.5 | BD Biosciences |
| CD11c | HL3 | BV421 | BD Biosciences |
| MHC II | M5/114.15.2 | Alexa700 | Biolegend |
| CD11b | M1/70 | APC-Cy7 | BD Biosciences |
| Siglec H | eBio440c | PE-Cy7 | Invitrogen |
| Ly6G | 1A8 | PE-CF594 | BD Biosciences |
| NK1.1 | PK136 | PE-CF594 | BD Biosciences |
| Siglec F | E50-2440 | PE-CF594 | BD Biosciences |
| B220 | RA3-6B2 | PE-CF594 | BD Biosciences |
| CD4 | RM4-5 | PE-CF594 | BD Biosciences |
| CD8 | 53-6.7 | PE-CF594 | BD Biosciences |
| GFAP | GA5 | efluor 660 | Invitrogen |

**Supplementary Table S2**. Analysis of differentially expressed genes in astrocytes after fingolimod treatment: Gene Ontology (GO) processes, pathways and genes with p<0.05.

| **GO Processes Enriched in Control Astrocytes (Downregulated with Fingolimod)** | **P-value** | **GO Processes Enriched in Fingolimod-Treated Astrocytes** | **P-value** |
| --- | --- | --- | --- |
| regulation of signal transduction (GO:0009966) * | 8.37E-04 | mitochondrion morphogenesis (GO:0070584) | 4.33E-03 |
| mitotic cell cycle phase transition (GO:0044772) | 5.57E-03 | histone H3-K4 trimethylation (GO:0080182) | 4.33E-03 |
| cell cycle G1/S phase transition (GO:0044843) | 5.62E-03 | dendrite morphogenesis (GO:0048813) | 4.43E-03 |
| intracellular protein transport (GO:0006886) | 8.09E-03 | negative regulation of chemotaxis (GO:0050922) * | 6.27E-03 |
| regulation of cell communication (GO:0010646) | 8.28E-03 | cell part morphogenesis (GO:0032990) | 6.27E-03 |
| positive regulation of vascular endothelial growth factor production (GO:0010575) | 8.97E-03 | attachment of mitotic spindle microtubules to kinetochore (GO:0051315) | 6.27E-03 |
| regulation of endodeoxyribonuclease activity (GO:0032071) | 9.97E-03 | atrial septum morphogenesis (GO:0060413) | 6.27E-03 |
| succinate metabolic process (GO:0006105) | 9.97E-03 | regulation of histone methylation (GO:0031060) | 7.36E-03 |
| regulation of protein import into nucleus (GO:0042306) | 0.010 | mitotic metaphase plate congression (GO:0007080) | 9.92E-03 |
| regulation of centrosome duplication (GO:0010824) | 0.010 | mesenchyme morphogenesis (GO:0072132) | 0.011 |
| 3'-phosphoadenosine 5'-phosphosulfate metabolic process (GO:0050427) | 0.010 | endocardial cushion morphogenesis (GO:0003203) | 0.011 |
| G1/S transition of mitotic cell cycle (GO:0000082) | 0.010 | mitochondrial translational termination (GO:0070126) | 0.013 |
| regulation of vascular endothelial growth factor production (GO:0010574) | 0.012 | peptidyl-lysine trimethylation (GO:0018023) | 0.014 |
| actin filament reorganization (GO:0090527) | 0.012 | translational termination (GO:0006415) | 0.017 |
| DNA damage response, signal transduction by p53 class mediator (GO:0030330) | 0.015 | positive regulation of cellular response to transforming growth factor beta stimulus (GO:1903846) | 0.017 |
| positive regulation of circadian rhythm (GO:0042753) | 0.015 | positive regulation of transforming growth factor beta receptor signaling pathway (GO:0030511) * | 0.017 |
| protein O-linked glycosylation (GO:0006493) | 0.016 | endocardial cushion development (GO:0003197) | 0.017 |
| cellular protein modification process (GO:0006464) | 0.016 | nucleotide-sugar biosynthetic process (GO:0009226) | 0.017 |
| regulation of cellular catabolic process (GO:0031329) | 0.016 | chordate embryonic development (GO:0043009) | 0.020 |
| autophagosome organization (GO:1905037) | 0.017 | mitochondrial fission (GO:0000266) | 0.021 |
| T-helper cell differentiation (GO:0042093) * | 0.018 | ubiquitin-dependent ERAD pathway (GO:0030433) | 0.022 |
| protein complex assembly (GO:0006461) | 0.018 | translational elongation (GO:0006414) | 0.023 |
| actin filament organization (GO:0007015) | 0.019 | regulation of extrinsic apoptotic signaling pathway (GO:2001236) | 0.023 |
| autophagosome assembly (GO:0000045) | 0.019 | central nervous system development (GO:0007417) | 0.024 |
| collagen metabolic process (GO:0032963) | 0.021 | mitochondrial translation (GO:0032543) | 0.024 |
| negative regulation of ERAD pathway (GO:1904293) | 0.021 | histone H3-K4 methylation (GO:0051568) | 0.024 |
| oligosaccharide biosynthetic process (GO:0009312) | 0.021 | positive regulation of histone methylation (GO:0031062) | 0.026 |
| establishment of chromosome localization (GO:0051303) | 0.024 | mitochondrion organization (GO:0007005) | 0.028 |
| prostanoid biosynthetic process (GO:0046457) | 0.024 | brain development (GO:0007420) | 0.034 |
| multivesicular body sorting pathway (GO:0071985) | 0.024 | aerobic respiration (GO:0009060) | 0.034 |
| copper ion homeostasis (GO:0055070) | 0.024 | organelle fission (GO:0048285) | 0.034 |
| atrial cardiac muscle cell action potential (GO:0086014) | 0.024 | regulation of lipid biosynthetic process (GO:0046890) | 0.034 |
| actin filament bundle organization (GO:0061572) | 0.024 | regulation of cell migration involved in sprouting angiogenesis (GO:0090049) | 0.039 |
| oligosaccharide metabolic process (GO:0009311) | 0.025 | positive regulation of insulin secretion (GO:0032024) | 0.041 |
| carbohydrate biosynthetic process (GO:0016051) | 0.026 | positive regulation of interleukin-1 beta production (GO:0032731) * | 0.041 |
| cellular response to decreased oxygen levels (GO:0036294) | 0.026 | negative regulation of extrinsic apoptotic signaling pathway (GO:2001237) | 0.043 |
| copper ion transport (GO:0006825) | 0.027 | protein glycosylation (GO:0006486) | 0.044 |
| positive regulation of protein modification by small protein conjugation or removal (GO:1903322) | 0.028 | ERAD pathway (GO:0036503) | 0.044 |
| actin filament bundle assembly (GO:0051017) | 0.028 | regulation of interleukin-12 production (GO:0032655) * | 0.046 |
| cellular response to hypoxia (GO:0071456) | 0.029 | neural tube closure (GO:0001843) | 0.046 |
| positive regulation of endothelial cell proliferation (GO:0001938) | 0.030 | axonemal dynein complex assembly (GO:0070286) | 0.046 |
| membrane repolarization during cardiac muscle cell action potential (GO:0086013) | 0.031 | regulation of postsynaptic membrane potential (GO:0060078) | 0.046 |
| regulation of centriole replication (GO:0046599) | 0.031 | chemical synaptic transmission, postsynaptic (GO:0099565) | 0.046 |
| pyrimidine-containing compound metabolic process (GO:0072527) | 0.031 | regulation of transforming growth factor beta receptor signaling pathway (GO:0017015) | 0.047 |
| mitochondrion localization (GO:0051646) | 0.031 | anaphase-promoting complex-dependent catabolic process (GO:0031145) | 0.047 |
| regulation of peptide secretion (GO:0002791) | 0.031 | positive regulation of cell differentiation (GO:0045597) | 0.048 |
| regulation of protein ubiquitination (GO:0031396) | 0.033 |  |  |
| organelle assembly (GO:0070925) | 0.033 |  |  |
| positive regulation of lymphocyte differentiation (GO:0045621) * | 0.034 |  |  |
| positive regulation of DNA-templated transcription, initiation (GO:2000144) | 0.034 |  |  |
| SCF-dependent proteasomal ubiquitin-dependent protein catabolic process (GO:0031146) | 0.038 |  |  |
| positive regulation of monocyte chemotaxis (GO:0090026) | 0.038 |  |  |
| regulation of interferon-gamma production (GO:0032649) * | 0.041 |  |  |
| response to hydrogen peroxide (GO:0042542) | 0.041 |  |  |
| membrane lipid metabolic process (GO:0006643) | 0.042 |  |  |
| prostaglandin biosynthetic process (GO:0001516) | 0.042 |  |  |
| regulation of cell motility (GO:2000145) | 0.043 |  |  |
| myeloid cell differentiation (GO:0030099) * | 0.043 |  |  |
| metaphase plate congression (GO:0051310) | 0.043 |  |  |
| negative regulation of phosphorylation (GO:0042326) | 0.043 |  |  |
| positive regulation of blood vessel endothelial cell migration (GO:0043536) | 0.045 |  |  |
| purine ribonucleoside bisphosphate metabolic process (GO:0034035) | 0.046 |  |  |
| protein heterotetramerization (GO:0051290) | 0.046 |  |  |
| regulation of catenin import into nucleus (GO:0035412) | 0.046 |  |  |
| cellular response to interleukin-7 (GO:0098761) | 0.046 |  |  |
| positive regulation of mononuclear cell migration (GO:0071677) | 0.046 |  |  |
| interleukin-7-mediated signaling pathway (GO:0038111) | 0.046 |  |  |
| post-translational protein modification (GO:0043687) | 0.049 |  |  |
| **GO Pathways Enriched in Control Astrocytes (Downregulated with Fingolimod)** | **P-value** | **GO Pathways Enriched in Fingolimod-Treated Astrocytes** | **P-value** |
| GDP-fucose biosynthesis_Homo sapiens_R-HSA-6787639 | 4.30E-03 | Mitochondrial translation termination_Homo sapiens_R-HSA-5419276 | 0.010 |
| Cell Cycle, Mitotic_Homo sapiens_R-HSA-69278 | 7.14E-03 | Mitochondrial translation_Homo sapiens_R-HSA-5368287 | 0.013 |
| RNA Polymerase III Transcription Initiation From Type 1 Promoter_Homo sapiens_R-HSA-76061 | 0.012 | Degradation of DVL_Homo sapiens_R-HSA-4641258 | 0.019 |
| Activation of Ca-permeable Kainate Receptor_Homo sapiens_R-HSA-451308 | 0.012 | SCF(Skp2)-mediated degradation of p27/p21_Homo sapiens_R-HSA-187577 | 0.022 |
| Ionotropic activity of Kainate Receptors_Homo sapiens_R-HSA-451306 | 0.012 | Diseases associated with the TLR signaling cascade_Homo sapiens_R-HSA-5602358 | 0.024 |
| Activation of Kainate Receptors upon glutamate binding_Homo sapiens_R-HSA-451326 | 0.015 | Diseases of Immune System_Homo sapiens_R-HSA-5260271 | 0.024 |
| Unwinding of DNA_Homo sapiens_R-HSA-176974 | 0.018 | Separation of Sister Chromatids_Homo sapiens_R-HSA-2467813 | 0.024 |
| TP53 Regulates Transcription of Death Receptors and Ligands_Homo sapiens_R-HSA-6803211 | 0.018 | Metabolism of proteins_Homo sapiens_R-HSA-392499 | 0.025 |
| SMAD2/SMAD3:SMAD4 heterotrimer regulates transcription_Homo sapiens_R-HSA-2173796 | 0.018 | Synthesis of substrates in N-glycan biosythesis_Homo sapiens_R-HSA-446219 | 0.026 |
| Detoxification of Reactive Oxygen Species_Homo sapiens_R-HSA-3299685 | 0.019 | Activation of NF-kappaB in B cells_Homo sapiens_R-HSA-1169091 | 0.029 |
| Cell Cycle_Homo sapiens_R-HSA-1640170 | 0.021 | Mitotic Anaphase_Homo sapiens_R-HSA-68882 | 0.031 |
| Interactions of Rev with host cellular proteins_Homo sapiens_R-HSA-177243 | 0.022 | Cyclin E associated events during G1/S transition_Homo sapiens_R-HSA-69202 | 0.032 |
| Regulation of necroptotic cell death_Homo sapiens_R-HSA-5675482 | 0.024 | TCR signaling_Homo sapiens_R-HSA-202403 | 0.032 |
| RNA Polymerase III Transcription Initiation_Homo sapiens_R-HSA-76046 | 0.024 | Mitotic Metaphase and Anaphase_Homo sapiens_R-HSA-2555396 | 0.032 |
| Cyclin D associated events in G1_Homo sapiens_R-HSA-69231 | 0.028 | Cyclin A:Cdk2-associated events at S phase entry_Homo sapiens_R-HSA-69656 | 0.033 |
| G1 Phase_Homo sapiens_R-HSA-69236 | 0.028 | APC/C:Cdh1 mediated degradation of Cdc20 and other APC/C:Cdh1 targeted proteins in late mitosis/early G1_Homo sapiens_R-HSA-174178 | 0.035 |
| MAP3K8 (TPL2)-dependent MAPK1/3 activation_Homo sapiens_R-HSA-5684264 | 0.031 | S Phase_Homo sapiens_R-HSA-69242 | 0.037 |
| Regulated Necrosis_Homo sapiens_R-HSA-5218859 | 0.031 | Signaling by TGF-beta Receptor Complex_Homo sapiens_R-HSA-170834 | 0.038 |
| RIPK1-mediated regulated necrosis_Homo sapiens_R-HSA-5213460 | 0.031 | Disassembly of the destruction complex and recruitment of AXIN to the membrane_Homo sapiens_R-HSA-4641262 | 0.039 |
| G2/M Transition_Homo sapiens_R-HSA-69275 | 0.032 | Signaling by Robo receptor_Homo sapiens_R-HSA-376176 | 0.041 |
| Mitotic G2-G2/M phases_Homo sapiens_R-HSA-453274 | 0.033 | SMAD2/SMAD3:SMAD4 heterotrimer regulates transcription_Homo sapiens_R-HSA-2173796 | 0.041 |
| RNA Polymerase III Abortive And Retractive Initiation_Homo sapiens_R-HSA-749476 | 0.034 | Biosynthesis of the N-glycan precursor (dolichol lipid-linked oligosaccharide, LLO) and transfer to a nascent protein_Homo sapiens_R-HSA-446193 | 0.044 |
| RNA Polymerase III Transcription_Homo sapiens_R-HSA-74158 | 0.034 | RIG-I/MDA5 mediated induction of IFN-alpha/beta pathways_Homo sapiens_R-HSA-168928 | 0.046 |
| Neurotransmitter Receptor Binding And Downstream Transmission In The Postsynaptic Cell_Homo sapiens_R-HSA-112314 | 0.038 | Negative regulators of RIG-I/MDA5 signaling_Homo sapiens_R-HSA-936440 | 0.046 |
| Cytosolic sulfonation of small molecules_Homo sapiens_R-HSA-156584 | 0.038 | Asparagine N-linked glycosylation_Homo sapiens_R-HSA-446203 | 0.048 |
| RNA Polymerase III Chain Elongation_Homo sapiens_R-HSA-73780 | 0.038 | Mitotic G1-G1/S phases_Homo sapiens_R-HSA-453279 | 0.049 |
| Transcriptional activity of SMAD2/SMAD3:SMAD4 heterotrimer_Homo sapiens_R-HSA-2173793 | 0.041 | Synthesis of GDP-mannose_Homo sapiens_R-HSA-446205 | 0.049 |
| RHO GTPases Activate Formins_Homo sapiens_R-HSA-5663220 | 0.049 | Negative regulation of TCF-dependent signaling by DVL-interacting proteins_Homo sapiens_R-HSA-5368598 | 0.049 |
| **Genes** **Enriched in Control Astrocytes (Downregulated with Fingolimod)** | **P-value** | **Genes Enriched in Fingolimod-Treated Astrocytes** | **P-value** |
| Gm38070 | pvalue | Mad1l1 | 1.37E-05 |
| Fitm2 | 2.21E-05 | Ppp1r13b | 2.29E-05 |
| Bpnt1 | 2.98E-05 | Gm37632 | 3.64E-05 |
| Gm24539 | 5.53E-05 | Synpr | 4.02E-05 |
| Gbp7 | 6.01E-05 | Thbs1 | 5.34E-05 |
| Ctif | 6.50E-05 | Slc19a2 | 2.28E-04 |
| Sap18 | 6.85E-05 | Grin2d | 3.63E-04 |
| E2f5 | 8.82E-05 | Zfp719 | 4.38E-04 |
| Iigp1 * | 9.32E-05 | Gm38366 | 4.60E-04 |
| Gbp4 | 9.38E-05 | Rpl13a | 6.68E-04 |
| Hmgb1 * | 1.11E-04 | 2700097O09Rik | 1.32E-03 |
| Rps12 | 1.14E-04 | Nfkbib * | 1.59E-03 |
| Snx2 | 1.34E-04 | Cachd1 | 2.19E-03 |
| Cmpk2 | 1.77E-04 | Tarbp1 | 2.50E-03 |
| Cys1 | 1.93E-04 | Abca3 | 2.52E-03 |
| Prkg2 | 2.35E-04 | Acy3 | 3.12E-03 |
| Hunk | 2.48E-04 | Mapk12 | 3.21E-03 |
| Gm24095 | 3.13E-04 | Gm37067 | 3.32E-03 |
| Zfp955a | 3.36E-04 | Dnah6 | 3.63E-03 |
| Mtmr1 | 3.94E-04 | Cdkn2b | 3.85E-03 |
| Mfsd10 | 4.12E-04 | Ctr9 | 3.93E-03 |
| Zfp1 | 4.20E-04 | Mtfr1 | 4.01E-03 |
| Nufip1 | 4.45E-04 | Sybu | 4.30E-03 |
| Gm4951 | 4.83E-04 | Jade3 | 4.48E-03 |
| Spata1 | 4.88E-04 | Zmat5 | 4.65E-03 |
| H2-K1 | 5.08E-04 | Skp2 | 4.70E-03 |
| Zfp770 | 5.10E-04 | Cfl2 | 4.73E-03 |
| Maml1 | 5.49E-04 | Ltc4s | 4.73E-03 |
| Nkiras2 | 5.89E-04 | Scarna2 | 4.97E-03 |
| Prdx3 | 6.19E-04 | 9930012K11Rik | 5.66E-03 |
| Pygo2 | 6.20E-04 | Gm7785 | 5.69E-03 |
| Zbed4 | 6.47E-04 | Gm38286 | 5.70E-03 |
| Rrm1 | 6.64E-04 | Nop58 | 5.80E-03 |
| Ccndbp1 | 6.89E-04 | Mrpl50 | 6.24E-03 |
| Slc25a20 | 7.17E-04 | Tnnt1 | 6.63E-03 |
| Gem | 8.54E-04 | Rrp8 | 7.24E-03 |
| AW112010 | 9.97E-04 | Apba3 | 7.29E-03 |
| E2f4 | 1.03E-03 | Msh5 | 7.45E-03 |
| Gm37138 | 1.09E-03 | Ints10 | 7.45E-03 |
| Pqlc2 | 1.27E-03 | Pthlh | 7.84E-03 |
| Zbtb5 | 1.37E-03 | Tmem167 | 7.86E-03 |
| Crtc2 | 1.38E-03 | St6galnac2 | 8.02E-03 |
| Mea1 | 1.54E-03 | Gm18406 | 8.34E-03 |
| Erg | 1.58E-03 | Lama2 | 8.40E-03 |
| Col1a1 | 1.61E-03 | Gabrg1 | 8.41E-03 |
| Tap1 | 1.74E-03 | Psmd12 | 8.54E-03 |
| Abcd3 | 1.86E-03 | Timm44 | 8.73E-03 |
| Rab3ip | 1.99E-03 | Dcaf5 | 9.11E-03 |
| Gtpbp3 | 2.01E-03 | Ankzf1 | 9.20E-03 |
| Ppie | 2.01E-03 | Pxdc1 | 9.24E-03 |
| Ccs | 2.12E-03 | Serpinb1b | 9.25E-03 |
| Srp9 | 2.21E-03 | Gm9881 | 9.41E-03 |
| Neil1 | 2.53E-03 | Esrp2 | 9.69E-03 |
| Mcm4 | 2.56E-03 | Map3k3 | 9.78E-03 |
| Gm12844 | 2.63E-03 | Gm37354 | 9.92E-03 |
| Zap70 | 2.71E-03 | Mgat3 | 0.010 |
| Tuba1a | 2.88E-03 | Tfcp2l1 | 0.010 |
| Gm17354 | 3.02E-03 | Gm37349 | 0.010 |
| Cstf3 | 3.14E-03 | Nsrp1 | 0.011 |
| Gm15421 | 3.24E-03 | Asnsd1 | 0.011 |
| Camsap3 | 3.46E-03 | Gm37366 | 0.011 |
| Tubgcp2 | 3.51E-03 | Snhg15 | 0.012 |
| Dok3 | 3.56E-03 | Mtrf1l | 0.012 |
| Slc12a7 | 3.61E-03 | Mrpl45 | 0.012 |
| Slc39a14 | 3.67E-03 | Clmn | 0.012 |
| A430110C17Rik | 3.70E-03 | Reln | 0.013 |
| Cxx1b | 4.20E-03 | Amz1 | 0.013 |
| Dscam | 4.31E-03 | Camta2 | 0.013 |
| B3gntl1 | 4.39E-03 | Traf3 | 0.013 |
| Igfbp3 | 4.51E-03 | AU020206 | 0.013 |
| Aqr | 4.69E-03 | Hgs | 0.014 |
| Cyp1b1 | 5.13E-03 | Magee1 | 0.014 |
| Brat1 | 5.21E-03 | Acvr1 | 0.014 |
| Lzts1 | 5.27E-03 | Pcyt1b | 0.014 |
| Ranbp1 | 5.34E-03 | Gm37531 | 0.015 |
| Slc35f5 | 5.54E-03 | Fkbp5 | 0.015 |
| Limd1 | 5.81E-03 | Plpp7 | 0.015 |
| Dgcr2 | 5.82E-03 | Psmc5 | 0.015 |
| Zfp521 | 5.86E-03 | AA474408 | 0.015 |
| Gemin2 | 6.05E-03 | Uncx | 0.016 |
| Gm6733 | 6.10E-03 | Dvl1 | 0.016 |
| Lrp12 | 6.30E-03 | Dhx34 | 0.016 |
| Dedd2 | 6.50E-03 | Efl1 | 0.016 |
| Llgl2 | 6.58E-03 | Ubn1 | 0.016 |
| Cpeb2 | 6.66E-03 | Mhrt | 0.016 |
| Stat5a | 6.77E-03 | Cd68 | 0.016 |
| B3galt5 | 6.86E-03 | Qpctl | 0.017 |
| Elp4 | 6.92E-03 | Slc35g1 | 0.017 |
| Rbp7 | 7.40E-03 | Zfp783 | 0.018 |
| Kat8 | 7.71E-03 | Inpp5d | 0.018 |
| Ginm1 | 7.75E-03 | Gm12166 | 0.018 |
| E430025E21Rik | 7.89E-03 | Sorbs1 | 0.018 |
| Gja4 | 8.03E-03 | Gm37396 | 0.018 |
| Pdcl3 | 8.04E-03 | Fem1b | 0.018 |
| Gm28792 | 8.16E-03 | 4930539J05Rik | 0.019 |
| Spsb1 | 8.37E-03 | Kirrel | 0.019 |
| Gm38057 | 8.51E-03 | Tsen15 | 0.019 |
| Tgds | 8.96E-03 | Higd2a | 0.019 |
| Tmem107 | 8.98E-03 | Mrpl30 | 0.020 |
| Sox12 | 8.99E-03 | Slc4a4 | 0.020 |
| Gramd3 | 9.11E-03 | Bambi | 0.020 |
| Slc2a12 | 9.28E-03 | Gm17249 | 0.020 |
| Sac3d1 | 9.30E-03 | 1110038F14Rik | 0.020 |
| Dck | 9.34E-03 | Vrk3 | 0.020 |
| Agmo | 9.40E-03 | Rogdi | 0.021 |
| Alas2 | 9.42E-03 | Myof | 0.021 |
| Tceal8 | 9.49E-03 | Edf1 | 0.022 |
| Plat | 9.67E-03 | Tcf19 | 0.022 |
| Gm20655 | 9.79E-03 | Pak3 | 0.022 |
| B3galt1 | 9.95E-03 | BC051142 | 0.024 |
| Coq6 | 0.010 | Robo2 | 0.025 |
| Tceal6 | 0.010 | Wdr82 | 0.025 |
| Spag4 | 0.011 | Col4a4 | 0.026 |
| Cep76 | 0.011 | Champ1 | 0.026 |
| Tap2 | 0.011 | Gm37644 | 0.026 |
| Trnau1ap | 0.011 | Car5b | 0.026 |
| L3mbtl3 | 0.011 | Gm20667 | 0.026 |
| 1700030K09Rik | 0.012 | Nubpl | 0.026 |
| Cd274 * | 0.012 | Gm25132 | 0.026 |
| Gm12892 | 0.012 | Rap1gap | 0.027 |
| Gng12 | 0.012 | Hmbox1 | 0.027 |
| Dlg3 | 0.012 | Gm38372 | 0.027 |
| Nrg3 | 0.012 | Cops2 | 0.027 |
| P3h2 | 0.012 | Map6 | 0.027 |
| Polr3a | 0.012 | Doc2b | 0.027 |
| Gsr | 0.012 | Gm13835 | 0.027 |
| Dirc2 | 0.012 | Deaf1 | 0.027 |
| Mmaa | 0.013 | Gm996 | 0.028 |
| Slc25a35 | 0.013 | Mpp6 | 0.028 |
| Fbxo6 | 0.013 | Gm15808 | 0.029 |
| Slfn10-ps | 0.013 | Nfyc | 0.029 |
| Gm13378 | 0.013 | Socs7 | 0.029 |
| Zfp956 | 0.013 | Mpi | 0.029 |
| Tspan3 | 0.013 | Atic | 0.029 |
| Trak1 | 0.014 | Sap130 | 0.029 |
| Cpne3 | 0.014 | Hapln2 | 0.029 |
| Cfap43 | 0.014 | Atg5 | 0.030 |
| Trpc3 | 0.014 | Mtdh | 0.030 |
| Lifr | 0.015 | 6030442K20Rik | 0.030 |
| Ccdc134 | 0.015 | Slc16a11 | 0.031 |
| Tpk1 | 0.015 | Abhd17a | 0.031 |
| Adcy3 | 0.015 | D2Wsu81e | 0.031 |
| Jagn1 | 0.015 | En2 | 0.033 |
| Gm28229 | 0.015 | Mogs | 0.033 |
| Mageh1 | 0.015 | Smu1 | 0.033 |
| Myg1 | 0.016 | F8 | 0.033 |
| Snx17 | 0.016 | Ppp2r5b | 0.034 |
| Mrps31 | 0.016 | Bhlhe41 | 0.034 |
| Paxip1 | 0.016 | Fam195a | 0.034 |
| Nsmce1 | 0.016 | Utp14b | 0.034 |
| Mettl3 | 0.016 | Gnpnat1 | 0.034 |
| Stam | 0.016 | Thrsp | 0.034 |
| Ift43 | 0.016 | Tet1 | 0.035 |
| Trappc13 | 0.016 | Arx | 0.035 |
| Gusb | 0.016 | Faim | 0.035 |
| Map2k4 | 0.016 | Snhg5 | 0.036 |
| Fbxo4 | 0.016 | Gtf2i | 0.036 |
| Zwilch | 0.016 | Uqcr10 | 0.036 |
| Crk | 0.016 | Hp1bp3 | 0.037 |
| Mb21d2 | 0.016 | Fam53a | 0.037 |
| Shroom1 | 0.016 | Isl1 | 0.037 |
| Cog5 | 0.016 | Syn3 | 0.037 |
| Psmb8 | 0.016 | Ccdc112 | 0.037 |
| Manba | 0.017 | Rrs1 | 0.037 |
| Setx | 0.017 | Arvcf | 0.037 |
| Zic5 | 0.017 | Slc35g2 | 0.038 |
| Banf1 | 0.018 | Sdhaf2 | 0.038 |
| Fryl | 0.018 | Gm12216 | 0.039 |
| Kdelr1 | 0.018 | Dbnl | 0.039 |
| Gbe1 | 0.018 | Fam183b | 0.039 |
| Rhoc | 0.018 | Stag2 | 0.039 |
| Gpc4 | 0.018 | Mff | 0.040 |
| Dnttip2 | 0.018 | Faf2 | 0.040 |
| Lcp1 | 0.018 | Morc2b | 0.041 |
| Mmp11 | 0.018 | BC005624 | 0.041 |
| Gm37593 | 0.018 | Gnb5 | 0.041 |
| Gm37645 | 0.019 | Hspa1a | 0.041 |
| Grip1 | 0.019 | Gm26800 | 0.041 |
| Irgm1 * | 0.019 | Gm28557 | 0.041 |
| Raf1 | 0.019 | Eya1 | 0.041 |
| Galnt4 | 0.019 | Etl4 | 0.041 |
| Gramd2 | 0.019 | Dnhd1 | 0.041 |
| Ociad2 | 0.019 | Lmo3 | 0.042 |
| Map1lc3a | 0.019 | Dgat2 | 0.042 |
| Usp33 | 0.019 | Heatr6 | 0.042 |
| 4933421O10Rik | 0.020 | Tgif2 * | 0.042 |
| 5430400D12Rik | 0.020 | Kcnk3 | 0.042 |
| Birc3 | 0.020 | Rgs6 | 0.042 |
| Stx12 | 0.020 | Lrrc75b | 0.043 |
| Csnk1a1 | 0.020 | Mphosph8 | 0.043 |
| Csf2ra | 0.020 | Mfap3l | 0.043 |
| Dnajc11 | 0.020 | Trim16 | 0.043 |
| Tmed4 | 0.020 | Gm14049 | 0.043 |
| 1700016K19Rik | 0.020 | Lgi3 | 0.044 |
| Zfp958 | 0.021 | Cse1l | 0.044 |
| Mettl4 | 0.021 | Bach2 | 0.045 |
| Sord | 0.021 | Fancg | 0.046 |
| Slc39a13 | 0.021 | Npas2 | 0.046 |
| Srgap2 | 0.021 | Gm15477 | 0.047 |
| Trafd1 | 0.021 | Rpl27a | 0.047 |
| Zfp53 | 0.021 | Ddx51 | 0.048 |
| Arhgef10l | 0.021 | Gprasp1 | 0.048 |
| Zfp458 | 0.021 | 1700086L19Rik | 0.048 |
| Rbbp5 | 0.022 | Kcne2 | 0.048 |
| Phpt1 | 0.022 | Angel1 | 0.049 |
| 2610524H06Rik | 0.022 | Greb1 | 0.049 |
| Vipr2 | 0.022 | Ccdc114 | 0.049 |
| Ano6 | 0.022 | R3hcc1 | 0.050 |
| Wbp1 | 0.023 | Pced1b | 0.050 |
| Brms1 | 0.023 |  |  |
| Rad21 | 0.023 |  |  |
| Armc10 | 0.023 |  |  |
| 2410004B18Rik | 0.024 |  |  |
| Tmem106a | 0.024 |  |  |
| Cdh9 | 0.024 |  |  |
| Ola1 | 0.024 |  |  |
| Npepl1 | 0.024 |  |  |
| H2-Ab1 | 0.024 |  |  |
| Pias2 | 0.025 |  |  |
| Papss1 | 0.025 |  |  |
| 4931428F04Rik | 0.025 |  |  |
| Ulk2 | 0.025 |  |  |
| Depdc7 | 0.025 |  |  |
| Cmc2 | 0.025 |  |  |
| Tceanc | 0.026 |  |  |
| Fbxw11 | 0.026 |  |  |
| Ilvbl | 0.026 |  |  |
| Cables2 | 0.026 |  |  |
| Mrpl34 | 0.026 |  |  |
| Tpst2 | 0.026 |  |  |
| Cdkn1a | 0.026 |  |  |
| Chmp5 | 0.027 |  |  |
| Fzd5 | 0.027 |  |  |
| Bysl | 0.027 |  |  |
| Hba-a1 | 0.027 |  |  |
| Atg4b | 0.028 |  |  |
| Prps2 | 0.028 |  |  |
| Ap3s1 | 0.028 |  |  |
| Evl | 0.028 |  |  |
| Epdr1 | 0.028 |  |  |
| Cfap46 | 0.028 |  |  |
| Mars2 | 0.028 |  |  |
| Gm6851 | 0.028 |  |  |
| Socs2 | 0.028 |  |  |
| Zdhhc6 | 0.029 |  |  |
| Slc35c1 | 0.029 |  |  |
| Efs | 0.029 |  |  |
| Kcnn2 | 0.029 |  |  |
| Pigyl | 0.029 |  |  |
| Gpank1 | 0.029 |  |  |
| Zmynd19 | 0.029 |  |  |
| Gbp3 | 0.029 |  |  |
| Fam189a2 | 0.030 |  |  |
| Rabl3 | 0.030 |  |  |
| Edem3 | 0.031 |  |  |
| Tnfrsf10b | 0.031 |  |  |
| Ube2j1 | 0.031 |  |  |
| Dlgap4 | 0.031 |  |  |
| Gm25409 | 0.031 |  |  |
| Gid4 | 0.033 |  |  |
| Gm15859 | 0.033 |  |  |
| Gm29055 | 0.033 |  |  |
| Suv39h2 | 0.033 |  |  |
| Ppp6c | 0.033 |  |  |
| Gm29019 | 0.034 |  |  |
| Colgalt2 | 0.034 |  |  |
| Ctdsp1 | 0.034 |  |  |
| Igtp | 0.035 |  |  |
| 9130019O22Rik | 0.035 |  |  |
| Tug1 | 0.035 |  |  |
| Rab27b | 0.035 |  |  |
| Gins3 | 0.035 |  |  |
| Gm12396 | 0.036 |  |  |
| Zfp598 | 0.036 |  |  |
| Fads3 | 0.036 |  |  |
| Cutc | 0.036 |  |  |
| Gm3307 | 0.036 |  |  |
| Commd1 | 0.036 |  |  |
| C1s1 | 0.036 |  |  |
| Rab3gap2 | 0.037 |  |  |
| Gtf3a | 0.037 |  |  |
| B2m | 0.037 |  |  |
| Gm38115 | 0.037 |  |  |
| Pik3c2b | 0.037 |  |  |
| Rcc1 | 0.038 |  |  |
| Fstl1 | 0.038 |  |  |
| Ttc7 | 0.039 |  |  |
| Ccdc12 | 0.039 |  |  |
| Ubxn1 | 0.039 |  |  |
| Polr3c | 0.039 |  |  |
| Col28a1 | 0.039 |  |  |
| C1qtnf1 | 0.040 |  |  |
| Mypop | 0.040 |  |  |
| C030005K15Rik | 0.040 |  |  |
| Pcdhgb8 | 0.040 |  |  |
| Uvssa | 0.040 |  |  |
| Gmds | 0.040 |  |  |
| Gm14878 | 0.041 |  |  |
| Pcmtd1 | 0.041 |  |  |
| Mdfic | 0.041 |  |  |
| Ppp2r3c | 0.041 |  |  |
| Ttc33 | 0.042 |  |  |
| B330016D10Rik | 0.042 |  |  |
| Neurl2 | 0.042 |  |  |
| Rtcb | 0.042 |  |  |
| Pcdhb16 | 0.042 |  |  |
| Gm3081 | 0.042 |  |  |
| Ppfibp1 | 0.042 |  |  |
| Pdlim7 | 0.043 |  |  |
| Lgals4 | 0.043 |  |  |
| Ptgs2 | 0.043 |  |  |
| Ccl11 | 0.043 |  |  |
| Rora | 0.044 |  |  |
| Abracl | 0.044 |  |  |
| 9330160F10Rik | 0.044 |  |  |
| Kcna5 | 0.045 |  |  |
| Phf8 | 0.045 |  |  |
| BC023829 | 0.045 |  |  |
| Mdk | 0.045 |  |  |
| Fam91a1 | 0.045 |  |  |
| Grid1 | 0.045 |  |  |
| Ypel1 | 0.045 |  |  |
| Crybb3 | 0.046 |  |  |
| 1700001L19Rik | 0.046 |  |  |
| Prep | 0.046 |  |  |
| Cd74 * | 0.046 |  |  |
| Gm36962 | 0.046 |  |  |
| Cenpq | 0.046 |  |  |
| Zcchc12 | 0.046 |  |  |
| Dact1 | 0.046 |  |  |
| Crim1 | 0.046 |  |  |
| Foxm1 | 0.046 |  |  |
| Ddx6 | 0.046 |  |  |
| Rnf44 | 0.047 |  |  |
| Ms4a6b | 0.047 |  |  |
| Gm6162 | 0.047 |  |  |
| Npm1 | 0.047 |  |  |
| Wwtr1 | 0.047 |  |  |
| B3galnt2 | 0.047 |  |  |
| Gpr173 | 0.047 |  |  |
| Prickle2 | 0.047 |  |  |
| Uqcc1 | 0.047 |  |  |
| Kremen1 | 0.047 |  |  |
| Fkbp1a | 0.048 |  |  |
| Bcl2l2 | 0.048 |  |  |
| Sdhaf3 | 0.048 |  |  |
| Gm10125 | 0.048 |  |  |
| Gm16089 | 0.048 |  |  |
| Tnpo1 | 0.048 |  |  |
| Slc30a10 | 0.048 |  |  |
| 3110043O21Rik | 0.049 |  |  |
| Grik4 | 0.049 |  |  |
| Swt1 | 0.049 |  |  |
| Zfp759 | 0.050 |  |  |
|  | 0.050 |  |  |

* Asterisks denote GO processes and pathways and genes discussed in the text.

**Supplementary Table S3.** Analysis of differentially expressed genes in endothelial cells after fingolimod treatment: Gene Ontology (GO) processes, pathways and genes with p<0.05.

| **GO Processes Enriched in Control Endothelial Cells (Downregulated with Fingolimod)** | **P-value** | **GO Processes Enriched in Fingolimod- Treated Endothelial Cells** | **P-value** |
| --- | --- | --- | --- |
| nucleotide-sugar biosynthetic process (GO:0009226) | 4.02E-03 | calcium ion transport into cytosol (GO:0060402) | 6.73E-04 |
| purine nucleotide metabolic process (GO:0006163) | 4.63E-03 | regulation of release of sequestered calcium ion into cytosol (GO:0051279) | 9.53E-04 |
| purine-containing compound biosynthetic process (GO:0072522) | 6.02E-03 | positive regulation of calcium ion transmembrane transport (GO:1904427) | 1.48E-03 |
| microtubule polymerization or depolymerization (GO:0031109) | 6.80E-03 | excitatory chemical synaptic transmission (GO:0098976) | 2.06E-03 |
| regulation of smooth muscle cell differentiation (GO:0051150) | 6.99E-03 | positive regulation of release of sequestered calcium ion into cytosol (GO:0051281) | 2.71E-03 |
| protein import into mitochondrial inner membrane (GO:0045039) | 6.99E-03 | cyclic-nucleotide-mediated signaling (GO:0019935) | 2.96E-03 |
| positive regulation of microtubule polymerization or depolymerization (GO:0031112) | 7.63E-03 | positive regulation of calcium ion transport into cytosol (GO:0010524) | 2.96E-03 |
| fatty acid alpha-oxidation (GO:0001561) | 8.89E-03 | regulation of cation channel activity (GO:2001257) | 3.67E-03 |
| protein targeting to vacuole (GO:0006623) | 0.010 | calcium ion transmembrane import into cytosol (GO:0097553) | 4.11E-03 |
| regulation of endothelial cell differentiation (GO:0045601) | 0.011 | cAMP-mediated signaling (GO:0019933) * | 4.45E-03 |
| action potential (GO:0001508) | 0.012 | response to cAMP (GO:0051591) | 5.50E-03 |
| tube closure (GO:0060606) | 0.013 | protein kinase C-activating G-protein coupled receptor signaling pathway (GO:0007205) * | 5.57E-03 |
| T cell homeostasis (GO:0043029) * | 0.013 | embryonic appendage morphogenesis (GO:0035113) | 6.46E-03 |
| positive regulation of transcription from RNA polymerase III promoter (GO:0045945) | 0.013 | regulation of calcineurin-NFAT signaling cascade (GO:0070884) | 7.42E-03 |
| histone H2B ubiquitination (GO:0033523) | 0.013 | establishment or maintenance of actin cytoskeleton polarity (GO:0030950) | 7.42E-03 |
| protein K11-linked deubiquitination (GO:0035871) | 0.013 | positive regulation of ion transport (GO:0043270) | 8.05E-03 |
| cellular iron ion homeostasis (GO:0006879) | 0.014 | positive regulation of potassium ion transport (GO:0043268) | 8.43E-03 |
| somite development (GO:0061053) | 0.016 | retinoic acid metabolic process (GO:0042573) | 8.43E-03 |
| neural tube closure (GO:0001843) | 0.018 | receptor localization to synapse (GO:0097120) | 0.012 |
| regulation of mesenchymal cell proliferation (GO:0010464) | 0.018 | regulation of gene silencing by RNA (GO:0060966) | 0.013 |
| regulation of vasculogenesis (GO:2001212) | 0.018 | regulation of posttranscriptional gene silencing (GO:0060147) | 0.013 |
| serine family amino acid biosynthetic process (GO:0009070) | 0.018 | limb morphogenesis (GO:0035108) | 0.016 |
| establishment of protein localization to mitochondrial membrane (GO:0090151) | 0.018 | regulation of actin filament-based process (GO:0032970) | 0.018 |
| cellular transition metal ion homeostasis (GO:0046916) | 0.019 | negative regulation of oxidative stress-induced cell death (GO:1903202) | 0.020 |
| diterpenoid metabolic process (GO:0016101) | 0.020 | regulation of AMPA receptor activity (GO:2000311) | 0.020 |
| iron ion homeostasis (GO:0055072) | 0.021 | calcium ion transport (GO:0006816) | 0.021 |
| negative regulation of protein kinase B signaling (GO:0051898) | 0.021 | regulation of ryanodine-sensitive calcium-release channel activity (GO:0060314) | 0.022 |
| nucleotide-sugar metabolic process (GO:0009225) | 0.021 | regulation of gene silencing by miRNA (GO:0060964) | 0.022 |
| steroid catabolic process (GO:0006706) | 0.021 | ventricular septum development (GO:0003281) | 0.026 |
| motile cilium assembly (GO:0044458) | 0.021 | regulation of actin cytoskeleton organization (GO:0032956) | 0.029 |
| acylglycerol metabolic process (GO:0006639) | 0.022 | cellular response to cAMP (GO:0071320) | 0.030 |
| purine ribonucleotide metabolic process (GO:0009150) | 0.024 | regulation of sodium ion transmembrane transport (GO:1902305) | 0.030 |
| copper ion transport (GO:0006825) | 0.024 | regulation of cation transmembrane transport (GO:1904062) | 0.030 |
| purine-containing compound catabolic process (GO:0072523) | 0.024 | regulation of cytosolic calcium ion concentration (GO:0051480) | 0.033 |
| purine-containing compound salvage (GO:0043101) | 0.024 | ceramide biosynthetic process (GO:0046513) | 0.034 |
| purine nucleotide catabolic process (GO:0006195) | 0.024 | regulation of potassium ion transmembrane transport (GO:1901379) | 0.034 |
| regulation of extrinsic apoptotic signaling pathway via death domain receptors (GO:1902041) | 0.027 | regulation of glutamate receptor signaling pathway (GO:1900449) | 0.034 |
| purine ribonucleoside monophosphate metabolic process (GO:0009167) | 0.027 | regulation of interleukin-12 production (GO:0032655) | 0.036 |
| embryonic cranial skeleton morphogenesis (GO:0048701) | 0.027 | positive regulation of calcium ion transport (GO:0051928) | 0.036 |
| microtubule depolymerization (GO:0007019) | 0.027 | regulation of neurotransmitter receptor activity (GO:0099601) | 0.036 |
| amide transport (GO:0042886) | 0.027 | embryonic limb morphogenesis (GO:0030326) | 0.038 |
| positive regulation of G1/S transition of mitotic cell cycle (GO:1900087) | 0.031 | actin filament bundle organization (GO:0061572) | 0.039 |
| positive regulation of leukocyte activation (GO:0002696) * | 0.031 | regulation of sodium ion transport (GO:0002028) | 0.039 |
| positive regulation of macrophage derived foam cell differentiation (GO:0010744) | 0.031 | calcium ion transmembrane transport (GO:0070588) | 0.041 |
| regulation of ion transmembrane transporter activity (GO:0032412) | 0.033 | glutamate receptor signaling pathway (GO:0007215) | 0.042 |
| chloride transport (GO:0006821) | 0.033 | actin filament bundle assembly (GO:0051017) | 0.044 |
| negative regulation of hydrolase activity (GO:0051346) | 0.033 | regulation of alternative mRNA splicing, via spliceosome (GO:0000381) | 0.048 |
| positive regulation of macrophage activation (GO:0043032) * | 0.034 | cognition (GO:0050890) | 0.050 |
| nucleobase-containing compound biosynthetic process (GO:0034654) | 0.034 |  |  |
| nucleotide catabolic process (GO:0009166) | 0.034 |  |  |
| regulation of Wnt signaling pathway (GO:0030111) * | 0.035 |  |  |
| protein transmembrane import into intracellular organelle (GO:0044743) | 0.035 |  |  |
| retinoid metabolic process (GO:0001523) | 0.036 |  |  |
| skeletal muscle contraction (GO:0003009) | 0.038 |  |  |
| negative regulation of microtubule depolymerization (GO:0007026) | 0.038 |  |  |
| sensory perception of mechanical stimulus (GO:0050954) | 0.041 |  |  |
| negative regulation of lymphocyte activation (GO:0051250) | 0.042 |  |  |
| protein targeting to lysosome (GO:0006622) | 0.042 |  |  |
| cellular response to heat (GO:0034605) | 0.044 |  |  |
| sensory perception of sound (GO:0007605) | 0.045 |  |  |
| negative regulation of protein depolymerization (GO:1901880) | 0.046 |  |  |
| regulation of fibroblast growth factor receptor signaling pathway (GO:0040036) * | 0.046 |  |  |
| regulation of macrophage activation (GO:0043030) | 0.050 |  |  |
| histone ubiquitination (GO:0016574) | 0.050 |  |  |
| ventricular cardiac muscle tissue development (GO:0003229) | 0.050 |  |  |
| **GO Pathways Enriched in Control Endothelial Cells (Downregulated with Fingolimod)** | **P-value** | **GO Pathways Enriched in Fingolimod- Treated Endothelial Cells** | **P-value** |
| Purine metabolism_Homo sapiens_R-HSA-73847 | 0.0002 | Activation of Ca-permeable Kainate Receptor_Homo sapiens_R-HSA-451308 | 3.27E-03 |
| Alpha-oxidation of phytanate_Homo sapiens_R-HSA-389599 | 0.0026 | Ionotropic activity of Kainate Receptors_Homo sapiens_R-HSA-451306 | 3.27E-03 |
| Abacavir metabolism_Homo sapiens_R-HSA-2161541 | 0.0026 | Transcriptional regulation by small RNAs_Homo sapiens_R-HSA-5578749 | 3.67E-03 |
| Purine catabolism_Homo sapiens_R-HSA-74259 | 0.0110 | Neurotransmitter Receptor Binding And Downstream Transmission In The Postsynaptic Cell_Homo sapiens_R-HSA-112314 | 8.29E-03 |
| Abacavir transport and metabolism_Homo sapiens_R-HSA-2161522 | 0.0110 | Ras activation uopn Ca2+ infux through NMDA receptor_Homo sapiens_R-HSA-442982 | 9.50E-03 |
| Metabolism of nucleotides_Homo sapiens_R-HSA-15869 | 0.0141 | Transmission across Chemical Synapses_Homo sapiens_R-HSA-112315 | 0.011 |
| Purine salvage_Homo sapiens_R-HSA-74217 | 0.0185 | Gene Silencing by RNA_Homo sapiens_R-HSA-211000 | 0.012 |
| Iron uptake and transport_Homo sapiens_R-HSA-917937 | 0.0330 | SALM protein interactions at the synapse_Homo sapiens_R-HSA-8849932 | 0.016 |
| Glucuronidation_Homo sapiens_R-HSA-156588 | 0.0417 | Neuronal System_Homo sapiens_R-HSA-112316 | 0.017 |
| TP53 Regulates Metabolic Genes_Homo sapiens_R-HSA-5628897 | 0.0489 | MicroRNA (miRNA) biogenesis_Homo sapiens_R-HSA-203927 | 0.017 |
| Mitophagy_Homo sapiens_R-HSA-5205647 | 0.0497 | CREB phosphorylation through the activation of Ras_Homo sapiens_R-HSA-442742 | 0.023 |
| Pink/Parkin Mediated Mitophagy_Homo sapiens_R-HSA-5205685 | 0.0497 | Activation of Kainate Receptors upon glutamate binding_Homo sapiens_R-HSA-451326 | 0.028 |
|  |  | Glutamate Binding, Activation of AMPA Receptors and Synaptic Plasticity_Homo sapiens_R-HSA-399721 | 0.030 |
|  |  | Trafficking of AMPA receptors_Homo sapiens_R-HSA-399719 | 0.030 |
|  |  | mRNA Splicing - Major Pathway_Homo sapiens_R-HSA-72163 | 0.030 |
|  |  | Post NMDA receptor activation events_Homo sapiens_R-HSA-438064 | 0.038 |
|  |  | mRNA Splicing_Homo sapiens_R-HSA-72172 | 0.038 |
|  |  | Activation of NMDA receptor upon glutamate binding and postsynaptic events_Homo sapiens_R-HSA-442755 | 0.046 |
|  |  | Elastic fibre formation_Homo sapiens_R-HSA-1566948 | 0.050 |
| **Genes Enriched in Control Endothelial Cells (Downregulated with Fingolimod)** | **P Value** | **Genes Enriched in Fingolimod-Treated Endothelial Cells** | **P Value** |
| Iigp1* | 2.53E-06 | Kcnb1 | 5.67E-07 |
| Ly6a | 7.57E-06 | Meg3 | 1.49E-04 |
| Irgm1* | 3.76E-05 | Fbln5 | 2.46E-04 |
| Gbp4 | 1.18E-04 | Hes7 | 2.72E-04 |
| Hba-a1 | 1.45E-04 | Zfp7 | 3.27E-04 |
| Ctla2a | 1.82E-04 | Mgp | 3.50E-04 |
| Hddc2 | 2.17E-04 | Cfap69 | 4.56E-04 |
| Rab11fip4 | 2.29E-04 | Eln | 5.81E-04 |
| Slc38a1 | 2.94E-04 | Olfr78 | 6.30E-04 |
| Ly6c1 | 3.52E-04 | Gkn3 | 6.39E-04 |
| Sele* | 3.54E-04 | Sox13 | 6.74E-04 |
| Scly | 3.71E-04 | Akap6 | 7.33E-04 |
| Gm12669 | 3.87E-04 | Neil1 | 1.04E-03 |
| Fnip1 | 3.90E-04 | Klhl29 | 1.24E-03 |
| Tgtp2 | 5.01E-04 | Nrp1 | 1.27E-03 |
| Nmb | 5.92E-04 | Polr2f | 1.47E-03 |
| B2m | 6.02E-04 | Plpp3 | 1.90E-03 |
| Timm10 | 6.37E-04 | Brd2 | 1.91E-03 |
| Akr1b10 | 6.80E-04 | Fam69b | 2.39E-03 |
| Fxyd5 | 6.90E-04 | Atp2b4 | 2.47E-03 |
| Nfatc2ip | 7.17E-04 | Gm17494 | 2.53E-03 |
| Sp5 | 7.40E-04 | Ltbp4 | 3.13E-03 |
| Rdh10 | 7.90E-04 | Fam107a | 3.71E-03 |
| Adal | 8.56E-04 | 5730522E02Rik | 3.78E-03 |
| Dgka | 8.93E-04 | Ppfia2 | 3.92E-03 |
| Cox11 | 1.08E-03 | Frrs1 | 4.24E-03 |
| Lysmd2 | 1.13E-03 | Ago1 | 4.65E-03 |
| Wnt5a | 1.14E-03 | Rian | 4.70E-03 |
| Tmem55a | 1.19E-03 | Map4k5 | 4.85E-03 |
| Hsf2 | 1.23E-03 | Dkk2 | 4.86E-03 |
| Diras2 | 1.26E-03 | Pde1a | 4.92E-03 |
| Rptor | 1.30E-03 | Rccd1 | 5.07E-03 |
| Cxcl12 | 1.34E-03 | Shisa6 | 5.34E-03 |
| Sema3c | 1.35E-03 | Dcdc2a | 5.93E-03 |
| Scube2 | 1.41E-03 | Camk2n1 | 5.95E-03 |
| Krt222 | 1.50E-03 | Nrep | 6.00E-03 |
| Arl2 | 1.55E-03 | Ccnd2 | 6.13E-03 |
| Zfp68 | 1.57E-03 | Gm26705 | 6.38E-03 |
| Sgk1 | 1.59E-03 | Gtf2ird2 | 6.53E-03 |
| Tmem252 | 1.66E-03 | Slc1a2 | 6.55E-03 |
| Astn1 | 1.69E-03 | Sfxn3 | 6.69E-03 |
| Amer1 | 1.83E-03 | Nfkbid * | 7.04E-03 |
| Gm12942 | 1.89E-03 | Oard1 | 7.30E-03 |
| H2-K1 | 1.90E-03 | Mycl | 7.41E-03 |
| Gm38299 | 1.95E-03 | Syngr3 | 7.54E-03 |
| Cep192 | 2.09E-03 | Fam57a | 7.71E-03 |
| Slc4a10 | 2.13E-03 | Itgb1 | 7.73E-03 |
| Stmn4 | 2.14E-03 | Bet1l | 7.99E-03 |
| Cyb5d2 | 2.36E-03 | Hexim1 | 8.41E-03 |
| Gbp2 | 2.51E-03 | Hbegf | 8.60E-03 |
| Ggh | 2.55E-03 | Zfp770 | 8.73E-03 |
| Thtpa | 2.65E-03 | Dlg1 | 8.99E-03 |
| Ndufaf4 | 2.96E-03 | Phlda1 | 9.08E-03 |
| Cpeb2 | 3.01E-03 | Acat1 | 9.11E-03 |
| Xpo4 | 3.09E-03 | Vegfc | 9.88E-03 |
| Med26 | 3.36E-03 | Slfn5 | 0.010 |
| Sema6b | 3.38E-03 | Tns2 | 0.011 |
| Pcdh7 | 3.39E-03 | Ptpn9 | 0.011 |
| Cep89 | 3.59E-03 | Tsga10 | 0.011 |
| Triqk | 3.73E-03 | 2310009B15Rik | 0.011 |
| Csnk1a1 | 3.73E-03 | Psmd4 | 0.011 |
| Mrpl22 | 3.76E-03 | Mmp25 | 0.012 |
| Galk2 | 3.77E-03 | Cited2 | 0.012 |
| Toe1 | 3.81E-03 | Cracr2a | 0.012 |
| Vti1a | 3.84E-03 | Col15a1 | 0.013 |
| Gbp3 | 4.16E-03 | Dram1 | 0.013 |
| Rgs20 | 4.40E-03 | Rbbp4 | 0.013 |
| Lrrtm2 | 4.41E-03 | 2900052N01Rik | 0.013 |
| Arfgef3 | 5.03E-03 | Rnd1 | 0.013 |
| Fam184a | 5.07E-03 | Ipo8 | 0.013 |
| Hbb-bs | 5.09E-03 | Zfp74 | 0.013 |
| Gm37183 | 5.23E-03 | Cir1 | 0.014 |
| Park2 | 5.25E-03 | Lamc1 | 0.014 |
| Celsr1 | 5.26E-03 | Zfp229 | 0.014 |
| Pikfyve | 5.38E-03 | Grin2b | 0.014 |
| Tap1 | 5.56E-03 | Atxn7l1 | 0.014 |
| H2-Eb1 | 5.59E-03 | Emp3 | 0.014 |
| Cnr1 | 5.64E-03 | Mfge8 | 0.014 |
| Ndufa4 | 5.66E-03 | Gm10320 | 0.014 |
| Atp7a | 5.85E-03 | Nes | 0.015 |
| Myh14 | 5.99E-03 | Rassf2 | 0.015 |
| Dnajc17 | 6.03E-03 | Grik5 | 0.015 |
| Fgfr2 | 6.23E-03 | Dsel | 0.015 |
| Rmnd5b | 6.60E-03 | 1700086O06Rik | 0.015 |
| Golga7b | 6.69E-03 | Eva1a | 0.015 |
| H2-DMb1 | 6.82E-03 | Fpgs | 0.016 |
| Serf1 | 7.03E-03 | Rnd3 | 0.016 |
| Spp1 | 7.06E-03 | Akip1 | 0.016 |
| Psmb8 | 7.06E-03 | Aplnr | 0.016 |
| Ywhaz | 7.15E-03 | Pdia4 | 0.016 |
| Xdh | 7.24E-03 | Epb41l2 | 0.017 |
| Olfm2 | 7.26E-03 | Igfbp3 | 0.017 |
| Atp2b3 | 7.67E-03 | Atf5 | 0.017 |
| Fth1 | 7.86E-03 | Cd24a | 0.017 |
| Pcdhga11 | 7.96E-03 | Map4k4 * | 0.018 |
| Rai2 | 8.28E-03 | Igfbp4 | 0.018 |
| Sorl1 | 8.30E-03 | Ppp1r15a | 0.018 |
| Sostdc1 | 8.52E-03 | Tshz1 | 0.018 |
| Tmem100 | 8.70E-03 | Rasgrf2 | 0.019 |
| Lrg1 | 8.72E-03 | Nsa2 | 0.019 |
| Gm37642 | 8.72E-03 | Srrt | 0.020 |
| Pmaip1 | 8.76E-03 | Stra6 | 0.020 |
| Nagpa | 8.78E-03 | Ufsp2 | 0.020 |
| Gm23751 | 8.95E-03 | Usp8 | 0.020 |
| Asl | 9.06E-03 | Abcb1b | 0.020 |
| Mal | 9.10E-03 | BC051226 | 0.020 |
| Lcn2 | 9.15E-03 | Rps11-ps1 | 0.020 |
| Ccdc126 | 9.17E-03 | Ier5l | 0.021 |
| Vps29 | 9.31E-03 | Sipa1l1 | 0.021 |
| Kif9 | 9.35E-03 | Crem | 0.021 |
| H2-T22 | 9.41E-03 | Gpr37 | 0.021 |
| Amdhd2 | 9.49E-03 | Gin1 | 0.022 |
| Cdc42ep5 | 9.57E-03 | Klc1 | 0.022 |
| Fgf13 | 9.73E-03 | Ptger1 | 0.022 |
| Chml | 9.80E-03 | Zeb2os | 0.022 |
| Prkch | 0.010 | Sugp1 | 0.022 |
| Alas2 | 0.010 | Hk1 | 0.023 |
| Katnb1 | 0.010 | Zfp704 | 0.023 |
| Adgra1 | 0.010 | Adgrv1 | 0.023 |
| 3110080O07Rik | 0.010 | Jakmip2 | 0.023 |
| Purg | 0.011 | Serpina1b | 0.023 |
| Cfap36 | 0.011 | Hist2h2be | 0.023 |
| Phyh | 0.011 | St6galnac6 | 0.024 |
| Rftn2 | 0.012 | Gm37874 | 0.024 |
| Zic3 | 0.012 | Runx1t1 | 0.024 |
| Sgta | 0.012 | Cdh23 | 0.024 |
| Rbm41 | 0.012 | Smpd4 | 0.025 |
| Slain2 | 0.012 | Mfap3l | 0.025 |
| Id1 | 0.012 | Josd2 | 0.025 |
| Zfp706 | 0.012 | Ahr | 0.025 |
| Syt2 | 0.012 | Nol3 | 0.026 |
| Celsr2 | 0.013 | Gins4 | 0.026 |
| Pts | 0.013 | Scara3 | 0.027 |
| Ywhah | 0.013 | Slc38a5 | 0.027 |
| Tpcn1 | 0.013 | Ets1 | 0.027 |
| Gm37522 | 0.013 | Cnbp | 0.027 |
| Snord89 | 0.013 | Dcaf10 | 0.027 |
| Dzank1 | 0.014 | Gm37534 | 0.027 |
| Plagl1 | 0.014 | Odf2l | 0.027 |
| Sowaha | 0.014 | Thoc3 | 0.027 |
| Itgb8 * | 0.014 | Gadd45b | 0.027 |
| H2-D1 | 0.014 | Rbm15 | 0.028 |
| Bsg | 0.014 | Cfap70 | 0.028 |
| Ppp1r21 | 0.014 | Rbm17 | 0.028 |
| A330009N23Rik | 0.014 | Upf2 | 0.028 |
| E2f4 | 0.014 | Gm16793 | 0.028 |
| Zfp870 | 0.015 | A830036E02Rik | 0.028 |
| Hbb-bt | 0.015 | Gm25410 | 0.029 |
| Orc4 | 0.015 | A630033H20Rik | 0.029 |
| Abcc4 | 0.015 | Uba2 | 0.029 |
| Usp54 | 0.015 | Fbxo18 | 0.029 |
| Fam118b | 0.016 | Rrad | 0.030 |
| Gtf2ird1 | 0.016 | Fbxw4 | 0.030 |
| Med12 | 0.016 | Actn4 | 0.030 |
| Rffl | 0.016 | Fam72a | 0.030 |
| Bag2 | 0.016 | Celf5 | 0.031 |
| Chrm1 | 0.016 | Crtc2 | 0.031 |
| Gm37452 | 0.016 | Tnk2 | 0.031 |
| Slc26a10 | 0.016 | Txnip | 0.031 |
| Plppr3 | 0.016 | Islr2 | 0.032 |
| Gm11772 | 0.016 | Cmklr1 | 0.032 |
| Zfp948 | 0.016 | Gm12294 | 0.033 |
| Zbtb42 | 0.017 | Ndufs4 | 0.033 |
| Bhlhb9 | 0.017 | Ccdc102a | 0.033 |
| Cecr2 | 0.017 | Tbl3 | 0.033 |
| Hhat | 0.017 | Bcor | 0.033 |
| Gmpr2 | 0.017 | Mast2 | 0.033 |
| Helq | 0.017 | Irf1 * | 0.034 |
| Tmem206 | 0.017 | Gm6863 | 0.034 |
| Usp45 | 0.017 | Cyp26b1 | 0.034 |
| Rlbp1 | 0.018 | Cenpb | 0.034 |
| Rrp12 | 0.018 | Rassf8 | 0.034 |
| Phactr3 | 0.018 | Sox6os | 0.034 |
| Rapgefl1 | 0.018 | Tars | 0.034 |
| Otub2 | 0.018 | Zfp319 | 0.034 |
| H2-Aa | 0.018 | Hmbox1 | 0.035 |
| Idua | 0.018 | Hipk3 | 0.035 |
| Sephs1 | 0.018 | Mus81 | 0.035 |
| Gemin8 | 0.018 | Arhgap23 | 0.035 |
| Nrm | 0.019 | Boc | 0.035 |
| Glul | 0.019 | Fzd10 | 0.035 |
| 9230114K14Rik | 0.019 | Gm18889 | 0.035 |
| 1700008J07Rik | 0.019 | Celf6 | 0.036 |
| Rnf20 | 0.019 | Bbs4 | 0.036 |
| Prrt1 | 0.019 | Fsbp | 0.036 |
| Mtmr10 | 0.019 | Ivns1abp | 0.036 |
| Utp15 | 0.020 | Dock8 | 0.036 |
| Ap1g2 | 0.020 | Ybx1 | 0.037 |
| Mllt3 | 0.020 | Gm28791 | 0.037 |
| Gm38355 | 0.020 | Rnf7 | 0.037 |
| Slc12a8 | 0.020 | Ptrf | 0.037 |
| Nek8 | 0.021 | Mrpl30 | 0.037 |
| AI838599 | 0.021 | Prrc1 | 0.037 |
| Cdk6 | 0.021 | S100a4 | 0.037 |
| Zfp334 | 0.021 | Arhgap39 | 0.037 |
| Rprd2 | 0.021 | Plekhh3 | 0.038 |
| Mfsd8 | 0.021 | Def8 | 0.039 |
| Sox17 | 0.021 | Tmem229a | 0.039 |
| Shisa4 | 0.021 | Abi3 | 0.039 |
| Eno1 | 0.022 | Cacng2 | 0.039 |
| Ercc6 | 0.022 | 4930500M09Rik | 0.039 |
| Tceal5 | 0.022 | Gm26621 | 0.040 |
| Lbhd1 | 0.022 | Ppp2r1a | 0.040 |
| Sfxn4 | 0.022 | Snord87 | 0.040 |
| Xrcc5 | 0.022 | Gm17971 | 0.040 |
| Mat2a | 0.022 | Stag1 | 0.040 |
| Rabl3 | 0.022 | Ftl1 | 0.040 |
| Wdr37 | 0.023 | Utp14b | 0.041 |
| Igtp | 0.023 | Srsf10 | 0.041 |
| Ddx52 | 0.023 | Sfxn1 | 0.041 |
| Aim1 | 0.023 | Cpsf2 | 0.042 |
| Tomm6 | 0.023 | Pank4 | 0.042 |
| Gm10039 | 0.023 | Jph4 | 0.042 |
| Mfsd4b4 | 0.023 | Ppm1l | 0.042 |
| Apold1 | 0.023 | Zfp935 | 0.042 |
| Isca2 | 0.024 | Utp11l | 0.042 |
| Zbtb44 | 0.024 | Cds2 | 0.042 |
| Slc11a2 | 0.024 | Zfhx3 | 0.042 |
| BC028528 | 0.024 | Ak3 | 0.043 |
| Gm9762 | 0.025 | Nudt4 | 0.043 |
| Ttc23 | 0.025 | Tmf1 | 0.043 |
| Gm24959 | 0.025 | Macf1 | 0.043 |
| Kif1bp | 0.025 | Chst11 | 0.043 |
| Mak16 | 0.025 | Aff1 | 0.043 |
| Tro | 0.025 | Gm14753 | 0.043 |
| Zc3h8 | 0.025 | Abhd16a | 0.043 |
| Armcx3 | 0.025 | Fbxl17 | 0.044 |
| Mrpl11 | 0.026 | Car4 | 0.044 |
| Pag1 | 0.026 | Hscb | 0.044 |
| Tmem245 | 0.026 | Rgs2 | 0.044 |
| Luc7l2 | 0.027 | Gm37716 | 0.045 |
| Asap3 | 0.027 | Cpne2 | 0.045 |
| Trub2 | 0.027 | Synpo | 0.045 |
| Zc3h7a | 0.027 | Hsp90aa1 | 0.046 |
| Fam65b | 0.027 | Swap70 | 0.046 |
| Slco1a4 | 0.027 | Specc1 | 0.046 |
| Tsc22d2 | 0.028 | Nrp2 | 0.046 |
| Rps12 | 0.028 | Ano1 | 0.046 |
| Ms4a4b | 0.028 | Ddx23 | 0.047 |
| Fam199x | 0.028 | Tspan6 | 0.047 |
| Thop1 | 0.028 | Rergl | 0.047 |
| Mapk4 | 0.028 | 4632415L05Rik | 0.048 |
| Alg1 | 0.028 | Zhx1 | 0.048 |
| Ranbp6 | 0.029 | Adh1 | 0.048 |
| Adh5 | 0.029 | Tceanc2 | 0.048 |
| Otud3 | 0.029 | Zmym6 | 0.048 |
| Tnni3 | 0.030 | Dgkh | 0.048 |
| Dstyk | 0.030 | Gm13536 | 0.048 |
| Mapk9 | 0.030 | Slk | 0.048 |
| Podn | 0.030 | Gm10125 | 0.049 |
| Hspa14 | 0.030 | Anpep | 0.049 |
| Hipk1 | 0.030 | Itfg2 | 0.049 |
| Dcaf6 | 0.030 | Mboat2 | 0.049 |
| Gm15445 | 0.030 | Pea15a | 0.049 |
| Dctn6 | 0.030 | Carhsp1 | 0.049 |
| Xpr1 | 0.030 | F2r | 0.049 |
| Dock1 | 0.031 | Afap1l2 | 0.050 |
| Nat8f1 | 0.031 | Dpf1 | 0.050 |
| F830016B08Rik | 0.031 | Arl6ip4 | 0.050 |
| Ppp2r3a | 0.031 | Olfml2b | 0.050 |
| Gm4673 | 0.031 | Pou3f4 | 0.050 |
| Nwd1 | 0.032 |  |  |
| Homer1 | 0.032 |  |  |
| Gm37283 | 0.032 |  |  |
| Hcar1 | 0.032 |  |  |
| 9430038I01Rik | 0.032 |  |  |
| Pgr | 0.032 |  |  |
| Notumos | 0.033 |  |  |
| Fnbp1l | 0.033 |  |  |
| Mypop | 0.033 |  |  |
| Stxbp4 | 0.033 |  |  |
| Gm12184 | 0.033 |  |  |
| Fbln1 | 0.033 |  |  |
| Cct5 | 0.033 |  |  |
| Rasgrp4 | 0.034 |  |  |
| Fam189b | 0.034 |  |  |
| Nub1 | 0.034 |  |  |
| N4bp3 | 0.034 |  |  |
| A630072M18Rik | 0.034 |  |  |
| Pcdhga8 | 0.034 |  |  |
| Zfp52 | 0.034 |  |  |
| Slc10a3 | 0.034 |  |  |
| Adss | 0.034 |  |  |
| C130071C03Rik | 0.034 |  |  |
| Rsph9 | 0.034 |  |  |
| Cldn11 | 0.035 |  |  |
| Zfp655 | 0.035 |  |  |
| Naaa | 0.035 |  |  |
| Rab22a | 0.035 |  |  |
| Wac | 0.035 |  |  |
| Elmod2 | 0.035 |  |  |
| Rrnad1 | 0.035 |  |  |
| Sumo1 | 0.035 |  |  |
| Gm10501 | 0.036 |  |  |
| Pard3 | 0.036 |  |  |
| Tceb2 | 0.036 |  |  |
| Mthfd1 | 0.036 |  |  |
| Gimap5 | 0.036 |  |  |
| Ablim2 | 0.036 |  |  |
| Gm26236 | 0.036 |  |  |
| Xrn1 | 0.037 |  |  |
| Ppid | 0.037 |  |  |
| Derl1 | 0.037 |  |  |
| BC024978 | 0.037 |  |  |
| Ugp2 | 0.037 |  |  |
| Mctp1 | 0.037 |  |  |
| Fam219b | 0.038 |  |  |
| Ttc12 | 0.038 |  |  |
| Nt5c2 | 0.038 |  |  |
| Rcn2 | 0.038 |  |  |
| Zbtb26 | 0.038 |  |  |
| Epha2 | 0.039 |  |  |
| Mcm2 | 0.039 |  |  |
| Ptgds | 0.039 |  |  |
| Tecr | 0.039 |  |  |
| Cdk10 | 0.039 |  |  |
| Sssca1 | 0.040 |  |  |
| Mitd1 | 0.040 |  |  |
| Agk | 0.040 |  |  |
| Mfsd7b | 0.040 |  |  |
| Phospho2 | 0.040 |  |  |
| Cyth2 | 0.040 |  |  |
| Macrod1 | 0.040 |  |  |
| Rnaseh2b | 0.040 |  |  |
| Syncrip | 0.040 |  |  |
| Cyb561d2 | 0.040 |  |  |
| Larp4b | 0.041 |  |  |
| Ensa | 0.041 |  |  |
| Fam161b | 0.041 |  |  |
| Gm12185 | 0.042 |  |  |
| Grpel2 | 0.042 |  |  |
| Lrrd1 | 0.042 |  |  |
| Fggy | 0.042 |  |  |
| Slc22a17 | 0.042 |  |  |
| Glmp | 0.042 |  |  |
| Dnali1 | 0.042 |  |  |
| Crym | 0.042 |  |  |
| Gm37393 | 0.042 |  |  |
| Gm5617 | 0.043 |  |  |
| Chrnb1 | 0.043 |  |  |
| Kcnq1 | 0.043 |  |  |
| D430019H16Rik | 0.043 |  |  |
| Lrrc8b | 0.043 |  |  |
| Stat2 | 0.044 |  |  |
| Fam214b | 0.044 |  |  |
| Herc4 | 0.044 |  |  |
| Efhc2 | 0.044 |  |  |
| Gm38235 | 0.044 |  |  |
| Folr1 | 0.044 |  |  |
| Gm7265 | 0.045 |  |  |
| Slc25a17 | 0.045 |  |  |
| Jmjd4 | 0.045 |  |  |
| Foxj3 | 0.045 |  |  |
| Lgals3 | 0.045 |  |  |
| Pno1 | 0.045 |  |  |
| Mrpl34 | 0.045 |  |  |
| Ppfia4 | 0.045 |  |  |
| Ccnl2 | 0.045 |  |  |
| Slc35e4 | 0.045 |  |  |
| Dbi | 0.046 |  |  |
| 1810043G02Rik | 0.046 |  |  |
| Gm21781 | 0.046 |  |  |
| Car12 | 0.046 |  |  |
| Dnajc28 | 0.046 |  |  |
| Lbp | 0.046 |  |  |
| Stk33 | 0.046 |  |  |
| Zfp931 | 0.046 |  |  |
| Pabpn1l | 0.046 |  |  |
| S1pr1 | 0.046 |  |  |
| Cdh12 | 0.046 |  |  |
| Vwa1 | 0.046 |  |  |
| Lpl | 0.047 |  |  |
| Scgb3a1 | 0.047 |  |  |
| Gabra4 | 0.047 |  |  |
| Ces2e | 0.047 |  |  |
| Tefm | 0.047 |  |  |
| Hace1 | 0.047 |  |  |
| Sik1 | 0.047 |  |  |
| Fancg | 0.047 |  |  |
| Smdt1 | 0.048 |  |  |
| Cobll1 | 0.048 |  |  |
| Klhl2 | 0.048 |  |  |
| Ankrd37 | 0.048 |  |  |
| Ydjc | 0.048 |  |  |
| Mllt4 | 0.049 |  |  |
| Wasl | 0.049 |  |  |
| Gm38352 | 0.049 |  |  |
| Apod | 0.049 |  |  |
| Psmb9 | 0.049 |  |  |
| Ugdh | 0.049 |  |  |
| Oscp1 | 0.049 |  |  |
| Slc35e2 | 0.050 |  |  |
| Gns | 0.050 |  |  |

* Asterisks denote GO processes and pathways and genes discussed in the text.

**Supplementary Table S4**. Analysis of differentially expressed genes in microglia after fingolimod treatment: Gene Ontology (GO) processes, pathways and genes with p<0.05.

| **GO Processes Enriched in Control Microglia (Downregulated with Fingolimod)** | **P-value** | **GO Processes Enriched in Fingolimod- Treated Microglia** | **P-value** |
| --- | --- | --- | --- |
| regulation of morphogenesis of an epithelium (GO:1905330) | 1.91E-03 | regulation of tube diameter (GO:0035296) | 5.88E-04 |
| regulation of organ morphogenesis (GO:2000027) | 1.91E-03 | myeloid leukocyte mediated immunity (GO:0002444) * | 9.40E-04 |
| fatty-acyl-CoA metabolic process (GO:0035337) | 7.71E-03 | regulation of blood vessel size (GO:0050880) | 1.61E-03 |
| peptidyl-tyrosine dephosphorylation (GO:0035335) | 0.013 | regulation of chemokine production (GO:0032642) * | 3.47E-03 |
| organophosphate ester transport (GO:0015748) | 0.013 | positive regulation of immunoglobulin production (GO:0002639) | 3.85E-03 |
| long-chain fatty-acyl-CoA metabolic process (GO:0035336) | 0.013 | positive regulation of lymphocyte proliferation (GO:0050671) | 4.60E-03 |
| modulation of excitatory postsynaptic potential (GO:0098815) | 0.013 | cAMP metabolic process (GO:0046058) | 5.99E-03 |
| negative regulation of cell-matrix adhesion (GO:0001953) | 0.015 | regulation of blood vessel diameter (GO:0097746) | 7.47E-03 |
| neuron projection extension (GO:1990138) | 0.016 | RNA phosphodiester bond hydrolysis (GO:0090501) | 8.00E-03 |
| regulation of postsynaptic membrane potential (GO:0060078) | 0.021 | positive regulation of B cell proliferation (GO:0030890) | 9.11E-03 |
| chemical synaptic transmission, postsynaptic (GO:0099565) | 0.021 | cyclic-nucleotide-mediated signaling (GO:0019935) | 0.010 |
| bone development (GO:0060348) | 0.023 | positive regulation of chemokine production (GO:0032722) | 0.012 |
| lipid transport (GO:0006869) | 0.024 | regulation of interleukin-6 production (GO:0032675) | 0.017 |
| regulation of mitochondrial membrane potential (GO:0051881) | 0.024 | regulation of B cell proliferation (GO:0030888) | 0.018 |
| negative regulation of cell-substrate adhesion (GO:0010812) | 0.024 | positive regulation of interleukin-6 production (GO:0032755) | 0.018 |
| regulation of muscle cell differentiation (GO:0051147) | 0.025 | positive regulation of B cell activation (GO:0050871) | 0.018 |
| regulation of ubiquitin protein ligase activity (GO:1904666) | 0.025 | positive regulation of macromolecule biosynthetic process (GO:0010557) | 0.029 |
| regulation of smoothened signaling pathway (GO:0008589) | 0.025 | organelle assembly (GO:0070925) | 0.031 |
| regulation of cell death (GO:0010941) | 0.027 | T-helper 17 cell lineage commitment (GO:0072540) | 0.032 |
| regulation of kinase activity (GO:0043549) | 0.031 | cellular response to monoamine stimulus (GO:0071868) | 0.032 |
| odontogenesis (GO:0042476) | 0.032 | hematopoietic stem cell differentiation (GO:0060218) | 0.032 |
| phosphate-containing compound metabolic process (GO:0006796) | 0.035 | negative regulation of bone resorption (GO:0045779) | 0.032 |
| regulation of synaptic transmission, glutamatergic (GO:0051966) | 0.038 | regulation of adiponectin secretion (GO:0070163) | 0.032 |
| telencephalon cell migration (GO:0022029) | 0.046 | lipoprotein catabolic process (GO:0042159) | 0.032 |
| regulation of MHC class I biosynthetic process (GO:0045343) | 0.046 | response to catecholamine (GO:0071869) | 0.032 |
| pyrimidine-containing compound transmembrane transport (GO:0072531) | 0.046 | positive regulation of cellular amide metabolic process (GO:0034250) | 0.034 |
| regulation of mitotic spindle checkpoint (GO:1903504) | 0.046 | regulation of toll-like receptor 9 signaling pathway (GO:0034163) * | 0.037 |
| regulation of respiratory system process (GO:0044065) | 0.046 | regulation of interleukin-18 production (GO:0032661) * | 0.037 |
| nucleobase metabolic process (GO:0009112) | 0.046 | regulation of chemokine biosynthetic process (GO:0045073) | 0.037 |
| regulation of cell cycle checkpoint (GO:1901976) | 0.046 | retinoic acid receptor signaling pathway (GO:0048384) | 0.037 |
| gland morphogenesis (GO:0022612) | 0.046 | protein maturation by iron-sulfur cluster transfer (GO:0097428) | 0.037 |
| negative regulation of type I interferon-mediated signaling pathway (GO:0060339) * | 0.046 | cAMP catabolic process (GO:0006198) | 0.037 |
| negative regulation of cell cycle phase transition (GO:1901988) | 0.046 | regulation of systemic arterial blood pressure by renin-angiotensin (GO:0003081) | 0.037 |
| response to manganese ion (GO:0010042) | 0.046 | negative regulation of neuron differentiation (GO:0045665) | 0.037 |
| regulation of NAD(P)H oxidase activity (GO:0033860) | 0.046 | cellular response to oxygen-containing compound (GO:1901701) | 0.039 |
| cellular triglyceride homeostasis (GO:0035356) | 0.046 | cGMP-mediated signaling (GO:0019934) | 0.041 |
| amine metabolic process (GO:0009308) | 0.046 | T-helper cell lineage commitment (GO:0002295) | 0.041 |
| renal filtration cell differentiation (GO:0061318) | 0.046 | regulation of response to oxidative stress (GO:1902882) | 0.041 |
| glomerular epithelial cell differentiation (GO:0072311) | 0.046 | regulation of T-helper 2 cell cytokine production (GO:2000551) | 0.041 |
| epithelial tube formation (GO:0072175) | 0.046 | cellular response to prostaglandin E stimulus (GO:0071380) | 0.041 |
| regulation of biosynthetic process (GO:0009889) | 0.046 | kinetochore assembly (GO:0051382) | 0.041 |
| positive regulation of nervous system development (GO:0051962) | 0.049 | negative regulation of bone remodeling (GO:0046851) | 0.041 |
| regulation of cell-matrix adhesion * (GO:0001952) | 0.049 | negative regulation of vascular permeability (GO:0043116) | 0.041 |
|  |  | plasma membrane to endosome transport (GO:0048227) | 0.041 |
|  |  | arachidonic acid secretion (GO:0050482) | 0.041 |
|  |  | regulation of cytoplasmic mRNA processing body assembly (GO:0010603) | 0.041 |
|  |  | lipoprotein localization (GO:0044872) | 0.041 |
|  |  | response to forskolin (GO:1904321) | 0.041 |
|  |  | arachidonate transport (GO:1903963) | 0.041 |
|  |  | positive regulation of behavior (GO:0048520) | 0.041 |
|  |  | regulation of brown fat cell differentiation (GO:0090335) | 0.041 |
|  |  | lipoprotein transport (GO:0042953) | 0.041 |
|  |  | cellular response to forskolin (GO:1904322) | 0.041 |
|  |  | galactose catabolic process (GO:0019388) | 0.041 |
|  |  | T-helper 17 cell differentiation (GO:0072539) | 0.041 |
|  |  | defense response to Gram-negative bacterium (GO:0050829) | 0.044 |
|  |  | protein polyglutamylation (GO:0018095) | 0.046 |
|  |  | positive regulation of production of molecular mediator of immune response (GO:0002702) | 0.046 |
|  |  | positive regulation of nitric-oxide synthase biosynthetic process (GO:0051770) | 0.046 |
|  |  | regulation of immunoglobulin secretion (GO:0051023) | 0.046 |
|  |  | oogenesis (GO:0048477) | 0.046 |
|  |  | positive regulation of immunoglobulin secretion (GO:0051024) | 0.046 |
|  |  | cyclic nucleotide catabolic process (GO:0009214) | 0.046 |
|  |  | negative regulation of lipid localization (GO:1905953) | 0.046 |
|  |  | hexose catabolic process (GO:0019320) | 0.046 |
|  |  | positive regulation of inflammatory response (GO:0050729) | 0.047 |
|  |  | cAMP-mediated signaling (GO:0019933) | 0.049 |
|  |  | leukotriene D4 biosynthetic process (GO:1901750) | 0.050 |
|  |  | I-kappaB phosphorylation (GO:0007252) | 0.050 |
|  |  | regulation of immunoglobulin production (GO:0002637) | 0.050 |
|  |  | RNA phosphodiester bond hydrolysis, endonucleolytic (GO:0090502) | 0.050 |
|  |  | galactose metabolic process (GO:0006012) | 0.050 |
|  |  | contractile actin filament bundle assembly (GO:0030038) | 0.050 |
|  |  | regulation of urine volume (GO:0035809) | 0.050 |
|  |  | positive regulation of type 2 immune response (GO:0002830) | 0.050 |
|  |  | negative regulation of sodium ion transmembrane transport (GO:1902306) | 0.050 |
|  |  | leukotriene D4 metabolic process (GO:1901748) | 0.050 |
|  |  | stress fiber assembly (GO:0043149) | 0.050 |
| **GO Pathways Enriched in Control Microglia (Downregulated with Fingolimod)** | **P-value** | **GO Pathways Enriched in Fingolimod- Treated Microglia** | **P-value** |
| Conversion from APC/C:Cdc20 to APC/C:Cdh1 in late anaphase_Homo sapiens_R-HSA-176407 | 6.97E-03 | Galactose catabolism_Homo sapiens_R-HSA-70370 | 0.023 |
| Inactivation of APC/C via direct inhibition of the APC/C complex_Homo sapiens_R-HSA-141430 | 7.71E-03 | Trafficking of myristoylated proteins to the cilium_Homo sapiens_R-HSA-5624138 | 0.023 |
| Inhibition of the proteolytic activity of APC/C required for the onset of anaphase by mitotic spindle checkpoint components_Homo sapiens_R-HSA-141405 | 7.71E-03 | Cargo trafficking to the periciliary membrane_Homo sapiens_R-HSA-5620920 | 0.024 |
| Mitotic Spindle Checkpoint_Homo sapiens_R-HSA-69618 | 8.49E-03 | Coenzyme A biosynthesis_Homo sapiens_R-HSA-196783 | 0.037 |
| APC/C:Cdc20 mediated degradation of Cyclin B_Homo sapiens_R-HSA-174048 | 0.010 | MAPK1 (ERK2) activation_Homo sapiens_R-HSA-112411 * | 0.041 |
| APC-Cdc20 mediated degradation of Nek2A_Homo sapiens_R-HSA-179409 | 0.012 | MAPK3 (ERK1) activation_Homo sapiens_R-HSA-110056 * | 0.046 |
| Sodium-coupled sulphate, di- and tri-carboxylate transporters_Homo sapiens_R-HSA-433137 | 0.033 | Adenylate cyclase activating pathway_Homo sapiens_R-HSA-170660 | 0.046 |
| Vitamin B2 (riboflavin) metabolism_Homo sapiens_R-HSA-196843 | 0.033 | Interleukin-6 signaling_Homo sapiens_R-HSA-1059683 * | 0.050 |
| Activation of C3 and C5_Homo sapiens_R-HSA-174577 | 0.039 | TRAF6 mediated IRF7 activation in TLR7/8 or 9 signaling_Homo sapiens_R-HSA-975110 | 0.050 |
| Phosphorylation of Emi1_Homo sapiens_R-HSA-176417 | 0.039 | Import of palmitoyl-CoA into the mitochondrial matrix_Homo sapiens_R-HSA-200425 | 0.050 |
| Acyl chain remodeling of DAG and TAG_Homo sapiens_R-HSA-1482883 | 0.046 |  |  |
| PTK6 promotes HIF1A stabilization_Homo sapiens_R-HSA-8857538 | 0.046 |  |  |
| **Genes Enriched in Control Microglia (Downregulated with Fingolimod)** | **P-value** | **Genes Enriched in Fingolimod- Treated Microglia** | **P-value** |
| Lrrc17 | 1.52E-05 | Tspan18 | 1.02E-05 |
| H2-K1 | 2.10E-05 | Unc93b1 | 1.18E-04 |
| Gls | 3.44E-05 | Atxn2l | 1.36E-04 |
| Ddx3x | 3.53E-05 | Fbxl12 | 1.52E-04 |
| Ttr | 4.00E-05 | Usp19 | 1.89E-04 |
| Ccnl1 | 4.85E-05 | Sec14l1 | 2.53E-04 |
| Plek | 5.55E-05 | Rab11fip3 | 2.96E-04 |
| Enpp2 | 6.53E-05 | Abl1 | 3.05E-04 |
| Ube2d3 | 7.46E-05 | Slco4a1 | 3.65E-04 |
| Nlrc5 | 1.28E-04 | Lrrc39 | 3.66E-04 |
| Actb | 1.72E-04 | Unc50 | 4.24E-04 |
| Skil | 2.67E-04 | Kctd13 | 4.66E-04 |
| Ubc | 4.08E-04 | Tmbim6 | 4.69E-04 |
| Cd74 | 4.10E-04 | Gnb2 | 4.97E-04 |
| Ubb | 4.20E-04 | Slc2a5 | 5.47E-04 |
| Jmjd1c | 4.78E-04 | Tnfrsf21 | 5.86E-04 |
| 2810013P06Rik | 4.92E-04 | Itgb2 | 7.90E-04 |
| Shroom1 | 5.44E-04 | Pigg | 8.09E-04 |
| Arid5a | 5.60E-04 | Sh3bp1 | 1.01E-03 |
| Zfp148 | 6.57E-04 | Angptl7 | 1.08E-03 |
| Neat1 | 6.77E-04 | Epb41l2 | 1.10E-03 |
| Snord89 | 7.63E-04 | Unc119b | 1.15E-03 |
| mt-Rnr1 | 9.84E-04 | Sec24c | 1.45E-03 |
| mt-Ta | 1.26E-03 | Acss1 | 1.48E-03 |
| Id2 | 1.44E-03 | Syvn1 | 1.59E-03 |
| mt-Tc | 1.46E-03 | Pan2 | 1.78E-03 |
| Maff | 1.64E-03 | Arnt | 1.80E-03 |
| Sub1 | 1.72E-03 | Arhgef2 | 1.83E-03 |
| Smad7 | 2.06E-03 | Ccdc6 | 2.38E-03 |
| Ms4a6c | 2.25E-03 | Cnot8 | 2.63E-03 |
| Ndufa10 | 2.33E-03 | Slc39a13 | 2.66E-03 |
| Ptprc | 2.49E-03 | Tsc22d4 | 2.66E-03 |
| Nampt | 2.50E-03 | Cd300c2 | 2.90E-03 |
| Eef1b2 | 2.59E-03 | Gm28792 | 2.94E-03 |
| Peli1 | 2.59E-03 | Mfsd4b4 | 3.01E-03 |
| Ier3 | 2.60E-03 | Lrp1 | 3.65E-03 |
| Prelid2 | 2.61E-03 | Snrnp70 | 3.71E-03 |
| Nxph1 | 2.81E-03 | Inafm2 | 3.71E-03 |
| Gpm6a | 2.83E-03 | Arid2 | 3.72E-03 |
| Mfsd9 | 3.02E-03 | Eva1a | 3.79E-03 |
| Npc2 | 3.30E-03 | Gm37402 | 4.08E-03 |
| Dock4 | 3.48E-03 | Mpzl3 | 4.08E-03 |
| Dnajb1 | 3.78E-03 | Adcy6 | 4.17E-03 |
| A330074H02Rik | 3.82E-03 | Mknk1 | 4.48E-03 |
| Pim1 | 3.99E-03 | Gtpbp2 | 4.53E-03 |
| Creb5 | 4.36E-03 | Cpt2 | 4.66E-03 |
| Hmgn1 | 4.56E-03 | Gtf3c4 | 4.81E-03 |
| Diaph2 | 4.58E-03 | Elovl5 | 4.82E-03 |
| Snora30 | 4.75E-03 | Ddx17 | 4.87E-03 |
| Vcam1 | 4.77E-03 | Prex1 | 4.90E-03 |
| Srsf7 | 5.09E-03 | Cfap20 | 4.96E-03 |
| Hsp90aa1 | 5.33E-03 | Usp8 | 5.21E-03 |
| Rtcb | 5.43E-03 | Cox17 | 5.26E-03 |
| Klf2 | 5.65E-03 | Itgam * | 5.34E-03 |
| Ifrd1 * | 5.82E-03 | Tmem86a | 5.36E-03 |
| Gm8995 | 5.83E-03 | Amotl1 | 5.41E-03 |
| Them4 | 6.06E-03 | Epha2 | 5.44E-03 |
| Wdr36 | 6.06E-03 | Fermt3 | 5.61E-03 |
| Tgoln1 | 6.11E-03 | Slc36a1 | 5.71E-03 |
| Fubp1 | 6.12E-03 | Cacna2d3 | 5.75E-03 |
| Gm37621 | 6.41E-03 | Pcsk7 | 6.15E-03 |
| Csnk1a1 | 6.73E-03 | Tdrd7 | 6.58E-03 |
| Col1a2 | 6.96E-03 | Acvr1 | 6.65E-03 |
| Gm5805 | 7.11E-03 | Col27a1 | 6.77E-03 |
| Slc9a9 | 7.12E-03 | Ppp1r12b | 7.06E-03 |
| Hint1 | 7.29E-03 | Fbrsl1 | 7.17E-03 |
| Cdk13 | 7.55E-03 | Cyfip1 | 7.58E-03 |
| Tsc22d2 | 7.66E-03 | Aamp | 7.59E-03 |
| Fam135a | 7.69E-03 | Ubqln1 | 7.60E-03 |
| Igfbp5 | 7.72E-03 | Edem2 | 7.71E-03 |
| Gm37759 | 7.80E-03 | Gm10324 | 7.83E-03 |
| Atf4 | 8.02E-03 | Nucb1 | 7.97E-03 |
| Csrnp1 | 8.10E-03 | Sema4c | 8.11E-03 |
| Gm15155 | 8.19E-03 | Pmpca | 8.63E-03 |
| Ndufaf6 | 8.23E-03 | Fnta | 8.65E-03 |
| Dusp5 | 8.25E-03 | Parp16 | 8.70E-03 |
| Hspa5 | 8.49E-03 | Cds2 | 8.73E-03 |
| Gm28707 | 8.62E-03 | Map4k2 | 9.03E-03 |
| Zfp655 | 8.91E-03 | Fndc5 | 9.12E-03 |
| Ptma | 8.97E-03 | Sh2b3 | 9.19E-03 |
| Cntn5 | 9.01E-03 | Abca1 | 9.72E-03 |
| Rps20 | 9.05E-03 | 1500002C15Rik | 9.75E-03 |
| Zfand5 | 9.23E-03 | Mafg | 9.75E-03 |
| Tcea1-ps1 | 9.30E-03 | Ppp1r15a | 9.93E-03 |
| Casp4 | 9.32E-03 | Faf2 | 0.010 |
| Etnk1 | 9.38E-03 | Sox13 | 0.010 |
| Nfkbiz | 9.43E-03 | Arhgef1 | 0.010 |
| Hnrnpu | 9.43E-03 | Rnf214 | 0.011 |
| Rfk | 9.56E-03 | Ano6 | 0.011 |
| Gm29488 | 9.65E-03 | Lrp5 | 0.011 |
| Mon1a | 9.84E-03 | Hsd17b4 | 0.011 |
| Ttc14 | 0.010 | Cmtm3 | 0.011 |
| Fgfr2 | 0.010 | Kif21b | 0.011 |
| Hspa8 | 0.010 | Plxnb2 | 0.011 |
| Clk1 | 0.010 | Nup88 | 0.011 |
| Eml6 | 0.011 | 9530068E07Rik | 0.011 |
| Vmp1 | 0.011 | Kbtbd12 | 0.012 |
| Nrip1 | 0.011 | Khsrp | 0.012 |
| Pcna | 0.011 | Sirpa | 0.012 |
| Ascc3 | 0.011 | Smurf1 | 0.012 |
| H3f3b | 0.011 | Scmh1 | 0.012 |
| Ptger4 | 0.011 | Ewsr1 | 0.013 |
| Cfap100 | 0.011 | Arhgef18 | 0.013 |
| mt-Rnr2 | 0.012 | Slc12a7 | 0.013 |
| Tlk2 | 0.012 | Tjp2 | 0.013 |
| Dgat2 | 0.012 | Arhgef6 | 0.014 |
| Fam173b | 0.012 | Rnf130 | 0.014 |
| Acer2 | 0.012 | Cbl | 0.014 |
| Lncpint | 0.012 | Jun | 0.014 |
| Gm15459 | 0.012 | Ccr5 | 0.014 |
| Nme4 | 0.013 | Alg12 | 0.014 |
| Nlgn3 | 0.013 | Zfp395 | 0.014 |
| Ptpn9 | 0.013 | Pbx2 | 0.015 |
| Hbb-bt | 0.013 | P4ha1 | 0.015 |
| Ctla2a | 0.013 | Pmpcb | 0.015 |
| AW554918 | 0.013 | Tbc1d20 | 0.015 |
| Laptm4b | 0.013 | Sh3pxd2a | 0.015 |
| Arid5b | 0.013 | Slc7a7 | 0.016 |
| 2610203C20Rik | 0.013 | Sh3gl1 | 0.016 |
| Med26 | 0.013 | Ctnnd1 | 0.016 |
| Prom1 | 0.014 | Cd38 | 0.016 |
| Lrrk2 | 0.014 | Pcyox1 | 0.016 |
| Hnrnpdl | 0.014 | Plekha2 | 0.017 |
| Atp6v1e1 | 0.014 | Rab23 | 0.017 |
| Sowahc | 0.014 | Tgfbr2 | 0.017 |
| Isoc1 | 0.014 | Acvr1b | 0.017 |
| Anapc15 | 0.014 | Akt1 | 0.017 |
| Gm28424 | 0.014 | Tmem184b | 0.017 |
| Gm37628 | 0.015 | Cds1 | 0.018 |
| Gm36638 | 0.015 | Atxn7l3b | 0.018 |
| Hspb8 | 0.016 | Rnf103 | 0.018 |
| Gm10138 | 0.016 | Flcn | 0.018 |
| Phf24 | 0.016 | Skiv2l | 0.019 |
| Atp7a | 0.016 | Dgcr6 | 0.019 |
| Ggnbp2 | 0.016 | Col15a1 | 0.019 |
| 2410080I02Rik | 0.016 | Nde1 | 0.019 |
| Prps1 | 0.016 | Cc2d1a | 0.019 |
| Gm37472 | 0.016 | Gzf1 | 0.019 |
| Ttll11 | 0.017 | Gale | 0.019 |
| Flrt3 | 0.017 | Pdp2 | 0.020 |
| Timp3 | 0.017 | Tollip | 0.020 |
| Atf5 | 0.017 | Llgl1 | 0.020 |
| Plk3 | 0.017 | Slc40a1 | 0.020 |
| Iigp1 * | 0.018 | Wdr5 | 0.021 |
| Nfkbib * | 0.018 | Cbx6 | 0.021 |
| Ptges3 | 0.018 | Gm15808 | 0.021 |
| Epha7 | 0.018 | Tchp | 0.021 |
| Fgl2 | 0.018 | 2700016F22Rik | 0.021 |
| Atraid | 0.019 | Icmt | 0.021 |
| Wdr45b | 0.019 | Rab8a | 0.021 |
| Cops4 | 0.019 | Commd1 | 0.021 |
| Ogfr | 0.019 | Hist1h3i | 0.022 |
| Cpsf6 | 0.019 | Aldh1b1 | 0.022 |
| Ajap1 | 0.019 | Gm37124 | 0.022 |
| Smchd1 | 0.019 | Slc6a6 | 0.022 |
| Atp6v1f | 0.020 | Tfcp2 | 0.022 |
| Pea15a | 0.020 | Ube2g2 | 0.022 |
| Tbx3os1 | 0.020 | Gcnt1 | 0.022 |
| Eif3h | 0.020 | Nudt9 | 0.023 |
| Mettl22 | 0.020 | Gm37010 | 0.023 |
| Scarna2 | 0.020 | Urb1 | 0.023 |
| Atp11b | 0.020 | Zc3h7b | 0.023 |
| Yme1l1 | 0.021 | Rusc2 | 0.023 |
| Glrx2 | 0.021 | Mtmr4 | 0.023 |
| Olfr46 | 0.021 | Brat1 | 0.023 |
| Cfdp1 | 0.021 | Zfp651 | 0.023 |
| Pou3f3 | 0.022 | Abcf2 | 0.023 |
| Mapk12 | 0.022 | Kcna6 | 0.024 |
| Ly6c1 | 0.022 | Stambp | 0.024 |
| Cd83 | 0.022 | Larp1 | 0.024 |
| Sfpq | 0.022 | Maf * | 0.024 |
| Tspo | 0.023 | Gm24492 | 0.025 |
| Rnps1 | 0.023 | Gm22980 | 0.025 |
| Arhgap15 | 0.023 | Uckl1 | 0.025 |
| Tmx1 | 0.023 | Gm26982 | 0.025 |
| Gm29291 | 0.024 | G0s2 | 0.025 |
| Cdc20 | 0.024 | Mid1ip1 | 0.026 |
| Gm37553 | 0.024 | Cbfb | 0.026 |
| Hmox1 | 0.024 | Toe1 | 0.026 |
| mt-Tp | 0.024 | Enthd2 | 0.026 |
| Sugt1 | 0.024 | Smarcc2 | 0.026 |
| Slc12a2 | 0.024 | Ccdc93 | 0.027 |
| Rprl3 | 0.025 | Csnk1d | 0.027 |
| Gm37818 | 0.025 | Lasp1 | 0.028 |
| Slc13a4 | 0.025 | Lace1 | 0.028 |
| Ythdc1 | 0.026 | Ccdc191 | 0.028 |
| Gm37738 | 0.026 | Lmo2 | 0.028 |
| Ifitm3 * | 0.026 | Glmp | 0.028 |
| Taf7 | 0.026 | Firre | 0.029 |
| Vps13c | 0.026 | Gmip | 0.029 |
| Znrf3 | 0.027 | Fbxo10 | 0.029 |
| Ddx5 | 0.027 | Sema4d | 0.029 |
| Zfp760 | 0.027 | Mtx3 | 0.029 |
| Ifi44 * | 0.027 | Snord7 | 0.030 |
| Trim30a | 0.028 | Irak2 | 0.030 |
| Rtn4 | 0.028 | 1810037I17Rik | 0.030 |
| Rps3 | 0.028 | Gm4707 | 0.030 |
| Gm5611 | 0.028 | Hcfc1 | 0.030 |
| Slc24a2 | 0.028 | Tlr9 * | 0.031 |
| 9430016H08Rik | 0.028 | Trip10 | 0.031 |
| Azin1 | 0.028 | Aldh3a2 | 0.031 |
| Pcdhga1 | 0.028 | 2310002F09Rik | 0.031 |
| Eif4a1 | 0.028 | Lin9 | 0.031 |
| 1110059E24Rik | 0.029 | C230037L18Rik | 0.031 |
| Sf3b1 | 0.029 | Acin1 | 0.031 |
| Gm13380 | 0.029 | Ran | 0.031 |
| Hspa14 | 0.029 | Ptrh2 | 0.031 |
| Gm8817 | 0.029 | Gm38178 | 0.032 |
| Gm12749 | 0.029 | Ppp1r8 | 0.032 |
| Mir142hg | 0.029 | Phrf1 | 0.032 |
| Setdb2 | 0.029 | Rnaseh1 | 0.032 |
| Nfkbid * | 0.030 | Dbt | 0.032 |
| Cep83 | 0.030 | Arsg | 0.032 |
| Gm20544 | 0.030 | Adgrg1 | 0.032 |
| Nfkbia | 0.031 | Plxdc1 | 0.032 |
| Gm37675 | 0.031 | Tgfbrap1 | 0.032 |
| 4930517O19Rik | 0.031 | Pank4 | 0.033 |
| Gm17690 | 0.031 | Mpp3 | 0.033 |
| Ar | 0.031 | Gm16286 | 0.033 |
| Faap24 | 0.031 | Dtnb | 0.034 |
| Vaultrc5 | 0.032 | Soga1 | 0.034 |
| Mcl1 | 0.032 | Abcd4 | 0.034 |
| C4b | 0.032 | Scamp5 | 0.034 |
| Gm15501 | 0.032 | Gm28438 | 0.034 |
| Txn2 | 0.032 | Snx19 | 0.034 |
| Mbnl1 | 0.032 | Tnfrsf1b | 0.034 |
| Gla | 0.032 | Ndst4 | 0.034 |
| Slfn5 | 0.033 | Kif3b | 0.034 |
| Evc | 0.033 | Trim68 | 0.035 |
| Gm38192 | 0.033 | Txndc5 | 0.035 |
| Supt4a | 0.033 | Rbm5 | 0.035 |
| Rel | 0.033 | Lcp1 | 0.035 |
| Gm12577 | 0.033 | Pld2 | 0.035 |
| Lcorl | 0.034 | Rundc3a | 0.035 |
| Mir5620 | 0.034 | Ace | 0.036 |
| Stk32a | 0.034 | Ripk2 | 0.036 |
| Med23 | 0.034 | Cmtm4 | 0.036 |
| Cox6a2 | 0.034 | Bmf | 0.037 |
| Gm17024 | 0.034 | Lmln | 0.037 |
| Ube2s | 0.034 | Fnbp1 | 0.037 |
| Suz12 | 0.034 | Ttyh3 | 0.037 |
| Gm21781 | 0.035 | Isg20l2 * | 0.037 |
| Trp53bp1 | 0.035 | Gmpr2 | 0.037 |
| Fam120aos | 0.035 | Adam1a | 0.037 |
| Cox8a | 0.035 | Sema4b | 0.037 |
| Tmem194 | 0.036 | Prkcd | 0.038 |
| Rcn1 | 0.036 | Mis12 | 0.038 |
| Snord49b | 0.036 | Il6 * | 0.038 |
| Fam120b | 0.037 | Ccdc58 | 0.038 |
| Ier2 | 0.037 | Ccl12 * | 0.038 |
| Aif1 | 0.037 | E430014B02Rik | 0.038 |
| Clic6 | 0.037 | Bfar | 0.038 |
| H2afj | 0.037 | Map2k1 | 0.038 |
| Car10 | 0.037 | Zmynd8 | 0.039 |
| Inhbb | 0.037 | Gm23969 | 0.039 |
| Gm37033 | 0.038 | Tbc1d19 | 0.039 |
| Uck1 | 0.038 | 9330162012Rik | 0.039 |
| Dleu2 | 0.038 | Coro1c | 0.040 |
| Rasa1 | 0.038 | Dus3l | 0.040 |
| Fbxl2 | 0.038 | Ltc4s | 0.040 |
| Kpna4 | 0.038 | Htra2 | 0.040 |
| Gm37084 | 0.039 | Zfp628 | 0.040 |
| Clpp | 0.039 | Sh2b1 | 0.040 |
| Rap1b | 0.039 | Dennd1a | 0.040 |
| Ier5 | 0.039 | Ldb1 | 0.040 |
| Mllt10 | 0.039 | Rad54l2 | 0.041 |
| Rpl10a | 0.040 | Tab2 | 0.041 |
| Gm38177 | 0.040 | Setdb1 | 0.041 |
| Tmem11 | 0.040 | Lrp12 | 0.041 |
| Fbxo3 | 0.040 | Lin52 | 0.041 |
| Etaa1 | 0.040 | Clec12a | 0.041 |
| Gm37563 | 0.040 | Peg12 | 0.041 |
| Rad21 | 0.040 | Rbm17 | 0.041 |
| Lix1 | 0.040 | Fcrls | 0.041 |
| 4930403P22Rik | 0.040 | Afg3l1 | 0.041 |
| Ighj1 | 0.040 | Emsy | 0.042 |
| Adamts17 | 0.041 | Ska1 | 0.042 |
| Gm11244 | 0.041 | Dpf2 | 0.042 |
| Ribc1 | 0.041 | Gnptab | 0.042 |
| Sirt3 | 0.041 | Trim8 | 0.042 |
| Nfyb | 0.041 | Pms2 | 0.042 |
| B230317F23Rik | 0.042 | Zfp157 | 0.042 |
| Nt5dc1 | 0.042 | Irf5 * | 0.043 |
| Tex10 | 0.042 | Rarb | 0.043 |
| Brinp2 | 0.043 | Selplg | 0.043 |
| Bcl6 | 0.043 | Banp | 0.043 |
| Tmem63a | 0.043 | Srsf4 | 0.043 |
| Sema6b | 0.043 | Cdc40 | 0.043 |
| Slfn9 | 0.044 | Gas6 | 0.043 |
| Elmod1 | 0.044 | Bola2 | 0.044 |
| Msh3 | 0.044 | Kmt5a | 0.044 |
| Ctse | 0.044 | Dlx1 | 0.044 |
| Tmem151a | 0.044 | Atxn1l | 0.044 |
| Bbip1 | 0.044 | Usp35 | 0.044 |
| Fam179b | 0.044 | Efnb1 | 0.045 |
| Rsph3a | 0.044 | Eif2ak4 | 0.045 |
| Sdccag8 | 0.044 | AI606181 | 0.045 |
| Herpud1 | 0.044 | Pgs1 | 0.046 |
| Parp8 | 0.044 | Crtc2 | 0.046 |
| Yipf1 | 0.044 | Tgfbr1 | 0.046 |
| Trim27 | 0.045 | Ccdc180 | 0.046 |
| Anp32b | 0.045 | Gm16072 | 0.046 |
| Hace1 | 0.045 | Kdm1a | 0.046 |
| Gm12174 | 0.045 | Sdhaf1 | 0.047 |
| Cxcl16 | 0.045 | Fbxl19 | 0.047 |
| Bcap29 | 0.045 | Zfp839 | 0.047 |
| Fndc3b | 0.046 | Arhgef40 | 0.047 |
| Hsph1 | 0.046 | Hfe | 0.047 |
| Kdm6a | 0.046 | Ints10 | 0.047 |
| Wisp1 | 0.046 | Cic | 0.047 |
| Gm37420 | 0.046 | Lyplal1 | 0.047 |
| Stxbp4 | 0.047 | Ggt7 | 0.048 |
| Slc25a33 | 0.047 | Zfp217 | 0.048 |
| Tra2a | 0.047 | Phtf1os | 0.048 |
| Larp4 | 0.047 | Fhl3 | 0.048 |
| Mllt4 | 0.047 | Mib2 | 0.049 |
| Gm37834 | 0.047 | Pde3a | 0.049 |
| Gm38073 | 0.047 | Zhx3 | 0.049 |
| Lat2 | 0.048 | 43170 | 0.049 |
| Dusp2 | 0.048 | Gm8451 | 0.049 |
| Gm5129 | 0.048 | Prpf40b | 0.049 |
| Gm13822 | 0.048 | Gid4 | 0.049 |
| Dynlrb1 | 0.049 | Tm7sf3 | 0.049 |
| Prpf31 | 0.049 | Dbx2 | 0.049 |
| Eogt | 0.049 | Vangl2 | 0.049 |
| Snord13 | 0.049 | Mlxip | 0.049 |
| Rgs10 | 0.049 | Piwil2 | 0.050 |
| Cox7a2l | 0.049 | Gm11423 | 0.050 |
| Pcdhgb2 | 0.049 | Ormdl1 | 0.050 |
| Tbr1 | 0.049 | Rassf8 | 0.050 |
| Gm24959 | 0.050 |  |  |
| Atp1b1 | 0.050 |  |  |
| Gm12631 | 0.050 |  |  |
| Mfsd1 | 0.050 |  |  |
| 5430420F09Rik | 0.050 |  |  |

* Asterisks denote GO processes and pathways and genes discussed in the text.
